# Experimental demonstration and pan-structurome prediction of climate-associated riboSNitches in *Arabidopsis*

**DOI:** 10.1186/s13059-022-02656-4

**Published:** 2022-04-19

**Authors:** Ángel Ferrero-Serrano, Megan M. Sylvia, Peter C. Forstmeier, Andrew J. Olson, Doreen Ware, Philip C. Bevilacqua, Sarah M. Assmann

**Affiliations:** 1 Department of Biology, Pennsylvania State University, University Park, State College, PA 16802 USA; 2 Department of Biochemistry, Microbiology, and Molecular Biology, Pennsylvania State University, University Park, State College, PA 16802 USA; 3 Cold Spring Harbor Laboratory, Cold Spring Harbor, NY 11724 USA; 4 USDA ARS NAA Robert W. Holley Center for Agriculture and Health, Ithaca, NY 14853 USA; 5 Department of Chemistry, Pennsylvania State University, University Park, State College, PA 16802 USA; 6 Center for RNA Molecular Biology, Pennsylvania State University, University Park, State College, PA 16802 USA

**Keywords:** *Arabidopsis thaliana*, CLIMtools, Genome-wide association study (GWAS), riboSNitch, Single nucleotide variant (SNV), Structurome, Transcriptome-wide association study (TWAS)

## Abstract

**Background:**

Genome-wide association studies (GWAS) aim to correlate phenotypic changes with genotypic variation. Upon transcription, single nucleotide variants (SNVs) may alter mRNA structure, with potential impacts on transcript stability, macromolecular interactions, and translation. However, plant genomes have not been assessed for the presence of these structure-altering polymorphisms or “riboSNitches.”

**Results:**

We experimentally demonstrate the presence of riboSNitches in transcripts of two *Arabidopsis* genes, *ZINC RIBBON 3* (*ZR3*) and *COTTON GOLGI-RELATED 3* (*CGR3*), which are associated with continentality and temperature variation in the natural environment. These riboSNitches are also associated with differences in the abundance of their respective transcripts, implying a role in regulating the gene's expression in adaptation to local climate conditions. We then computationally predict riboSNitches transcriptome-wide in mRNAs of 879 naturally inbred *Arabidopsis* accessions. We characterize correlations between SNPs/riboSNitches in these accessions and 434 climate descriptors of their local environments, suggesting a role of these variants in local adaptation. We integrate this information in CLIMtools V2.0 and provide a new web resource, T-CLIM, that reveals associations between transcript abundance variation and local environmental variation.

**Conclusion:**

We functionally validate two plant riboSNitches and, for the first time, demonstrate riboSNitch conditionality dependent on temperature, coining the term “conditional riboSNitch.” We provide the first pan-genome-wide prediction of riboSNitches in plants. We expand our previous CLIMtools web resource with riboSNitch information and with 1868 additional *Arabidopsis* genomes and 269 additional climate conditions, which will greatly facilitate in silico studies of natural genetic variation, its phenotypic consequences, and its role in local adaptation.

**Supplementary Information:**

The online version contains supplementary material available at 10.1186/s13059-022-02656-4.

## Background

Natural genetic variation can give rise to variation in proximate molecular phenotypes that impact macroscopic physiological and morphological phenotypes. Perhaps the most obvious proximate molecular phenotype consists of alterations in protein sequence that arise from non-synonymous single nucleotide polymorphisms (SNPs), often with functional consequences. More recently, the influence of synonymous variants, which by definition do not alter the sequence of the encoded protein, has been increasingly appreciated, with one meta-analysis showing equal association of synonymous and non-synonymous mutations with human genetic disease [1].

One mechanism by which synonymous SNPs, as well as non-synonymous SNPs and SNPs located in introns and UTRs, can play regulatory roles is by changing the structure of the RNA in which they reside. Variants that alter RNA structure have been named riboSNitches by Laederach and colleagues [2], a term that is a portmanteau of “SNP” (single nucleotide polymorphism) and “riboswitch,” with the latter term referring to an RNA that changes conformation upon ligand binding [3].

Altered mRNA folding can impact mRNA splicing [4], mRNA turnover [5–7], mRNA processing [8, 9], mRNA intermolecular interactions with other RNAs [10–12] and with RNA-binding proteins [4, 9, 13, 14], and translation [15–20]. However, identification of riboSNitches is considerably more challenging than the identification of synonymous and non-synonymous variants, both of which can be deduced from sequence alone. In 1999, in one of the earliest identifications of a riboSNitch, Stanton and colleagues employed a nuclease that specifically cleaves single-stranded RNA and demonstrated allele-specific differential cleavage in the mRNAs encoding human alanyl tRNA synthetase and a replication protein A subunit [21]. By 2013, at least 30 disease-associated candidate riboSNitches had been identified [22]. However, of these, only four were designated as experimentally confirmed riboSNitches; the rest were only computationally predicted, emphasizing the challenges of experimental riboSNitch identification. This difficulty has been partially allayed by the recent coupling of RNA structure-probing methods [23] with high-throughput sequencing to provide mRNA structural predictions transcriptome-wide [24–26]. Using one such method, Wan, Chang and colleagues performed nuclease-based structure probing of ex vivo isolates from lymphoblastoid cells to survey the structuromes of a mother, father, and child trio [27]. They found that 1900 SNVs, comprising approximately 15% of transcript SNPs, were associated with differing structural signatures. Of these, nine were confirmed by an orthogonal structure-probing method. Among the 1900 SNVs, 211 were associated with alterations in the transcript's levels by eQTL analysis, while 22 were present in the NHGRI database of SNPs that have been associated with disease states by GWA analyses [27].

The groundbreaking analyses of Wan et al. point to the importance of riboSNitches in the control of gene expression, with fundamental whole-organism consequences. However, this study could obviously only evaluate those variants present in this family trio and so could not encompass the breadth of variation present in the multitude of human genome sequences currently available. Moreover, to date, this analysis of three individual humans remains the only large-scale experimental study of mammalian riboSNitches. Thus, the field still relies primarily on computational riboSNitch prediction. Leading riboSNitch prediction algorithms SNPfold [2], RNAsnp [22], and remuRNA [28] employ various metrics to identify statistically significant differences in local base-pairing probabilities in ensembles of structures arising from reference vs. alternative sequences. An evaluation of riboSNitch prediction methods by the Laederach group [29] confirmed that such algorithms perform better than more general RNA structure prediction methods at correctly identifying the orthogonally verified riboSNitches of Wan et al. [27]. A similar ensemble-based approach is employed in Riprap from the Ouyang lab [30]. Furthermore, Ouyang and colleagues provide a database, RiboSNitchDB, which collates in silico predicted riboSNitches in humans associated with previously identified eQTLs, as well as riboSNitches from Wan et al.’s study.

By contrast, to our knowledge, there have been no riboSNitches identified in plants by either wet bench or computational approaches. *Arabidopsis thaliana* (*Arabidopsis*) is a premier model plant species in molecular genetics, ecology, and evolutionary biology [31–35]. *Arabidopsis* is naturally inbreeding, and the resultant homozygosity facilitates its use in association studies that seek to correlate genetic and phenotypic variation and thereby identify causative genes. Here, we rectify the absence of plant riboSNitch information. We first confirm the existence of climate-related riboSNitches through wet bench analyses. We then employ SNPfold to predict *Arabidopsis* riboSNitches across 879 *Arabidopsis* genomes, evaluate global properties of these riboSNitches, and incorporate this information in CLIMtools.

Because plants are sessile, they adapt to local climate conditions, and resulting signatures of selection can be observed as statistical associations between variation in climate parameters and natural genetic variation. Accordingly, we previously assembled CLIMtools V1 (http://www.CLIMtools.org) [36], a web resource in which the user can identify the association of promoter and transcript variants with any of 204 geo-environmental variables, collated in our AraCLIM tool, that characterize the known collection sites of fully sequenced *Arabidopsis* accessions [36]. Users can query for climate-gene associations based on any *Arabidopsis* gene of interest (GenoCLIM) or based on any climate parameter of interest (CLIMGeno). Here, we expand the CLIMtools web resource through the inclusion of information on genome-wide riboSNitch prediction. We develop a new RNA-related tool, T-CLIM, which for the first time allows the community to query correlations between available RNA-seq data for 558 Eurasian accessions [37] and climate variables pan-transcriptome-wide. Moreover, our entire database is expanded through the inclusion of 1868 additional geo-located *Arabidopsis* accessions with available whole-genome sequence information and 269 additional climate parameters.

## Results

### Identification of candidate climate-associated riboSNitches

**Fig. 1:**
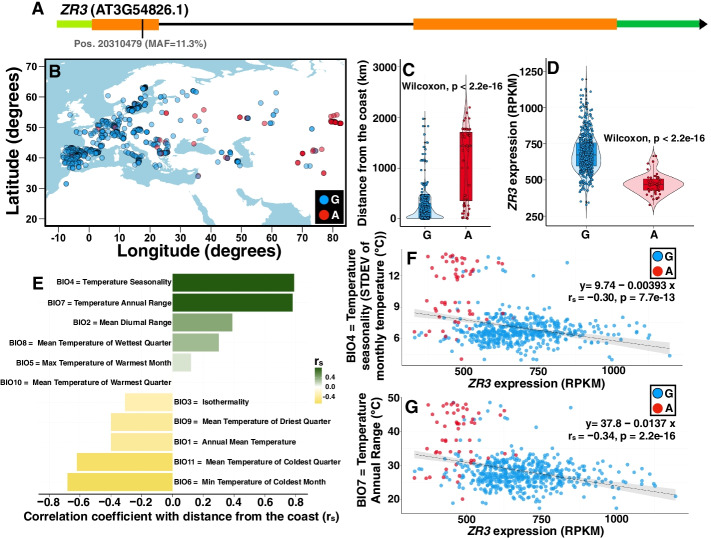
Allele distribution in a synonymous SNP in *ZR3* and its *cis*-regulated transcript abundance are correlated with the distance from the coast and temperature variability. **A** We explored an environmental cline in a SNP with a synonymous effect in *ZR3* (position chr3:20310479). Green indicates UTRs, orange indicates exons, and black indicates the sole intron. **B** The map of Eurasia shows the geographical distribution of the allelic variants of this SNP. Blue dots show the distribution of accessions with the major allele (“G,” for guanine), while red dots show the geographical distribution of accessions harboring the minor allele (“A,” for adenine). **C** Given the geographic distribution of both alleles, the probability of encountering an accession with the minor allele increases in accessions more distant from the coastline. **D** Violin plots illustrating significantly different probability densities of *ZR3* transcript abundance for the major and minor alleles of the SNP depicted in A. **E** Distance from the coast determines the temperature variability that accessions encounter in their local environment. **F**, **G** As plants endure a higher degree of temperature variability inland (*y*-axis in **F** and **G**), the probability of harboring a minor allele (red dots) at site 20,310,479 increases. At the same time, the transcript abundance of *ZR3* (*x*-axis in **F** and **G**) decreases, highlighting the regulatory effect of this SNP and its correlation with temperature variability. The regression is calculated using the transcript abundance data in the combined set of accessions (both major and minor alleles). Distance from the coast was derived from the NASA Ocean Biology processing group dataset. Climate variables are from the WorldClim 2.1 database. MAF, minor allele frequency; STDEV, standard deviation

**Fig. 2:**
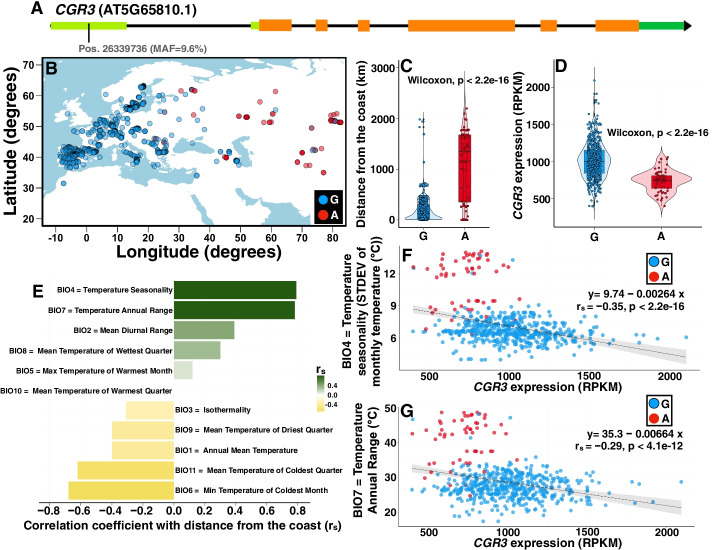
Allele distribution in a SNP in the 5′ UTR of *CGR3* and its *cis*-regulated transcript abundance are correlated with the distance from the coast and temperature variability. **A** We explored an environmental cline in a SNP in the 5′ UTR of *CGR3* (position chr5:26339736). Green indicates UTRs, orange indicates exons, and black indicates introns. **B** The map of Eurasia shows the geographical distribution of the allelic variants of this SNP. Blue dots show the distribution of accessions with the major allele (G), while red dots show the geographical distribution of accessions harboring the minor allele (A). **C** Given the geographic distribution of both alleles, the probability of encountering an accession with the minor allele increases in accessions more distant from the coastline. **D** Violin plots illustrating significantly different probability densities of *CGR3* transcript abundance for the major and minor alleles of the SNP depicted in **A**. **E** Distance from the coast determines the temperature variability that accessions encounter in their local environment. (Note that these are the same data as in Fig. 1E, reproduced here for ease of comparison with the rest of Fig. 2). **F**, **G** As plants endure a higher degree of temperature variability inland (*y*-axis in **F** and **G**), the probability of harboring a minor allele (red dots) at site 26,339,736 increases. At the same time, the transcript abundance of *CGR3* (*x*-axis in **F** and **G**) decreases, highlighting the regulatory effect of this SNP and its correlation with temperature variability. The regression is calculated using the transcript abundance data in the combined set of accessions (both major and minor alleles). Distance from the coast was derived from the NASA Ocean Biology processing group dataset. Climate variables are from the WorldClim 2.1 database. MAF, minor allele frequency; STDEV, standard deviatioon

In order to identify putative riboSNitches that are significantly associated with climate for detailed wet bench analyses, we first identified in CLIMtools SNPs that are associated with one or more of 48 temperature-related climate variables (Additional file 1: Table S1), and for which variation in transcript abundance is associated with the same climate variable (see the “Materials and methods” section for details). The resulting subset (Additional file 2: Table S2) included strong candidates with adaptive value to temperature. We then used SNPfold to determine which of these were potential riboSNitches (Additional file 3: Table S3), resulting in the identification of 13 single nucleotide variants (SNVs). For each of these, the structures of full-length mRNAs differing only by the reference and alternative SNP were predicted by a minimal free energy method using the RNAstructure software package [38]. In particular, AT3G54826 (*ZR3*) and AT5G65810 (*CGR3*) were predicted to encompass local secondary structure surrounding the SNP, as expected for genuine riboSNitches. In *ZR3*, the variant is a synonymous substitution in the coding sequence while in *CGR3*, the variant is located in the 5′ UTR (Figs. 1A and 2A). In further analysis of these two genes, we confirmed the climate and transcript-abundance association of their variants (Figs. 1 and 2). We found that *Arabidopsis* accessions resident in geographic locations further from the coast (Figs. 1B, C and 2B, C) are more likely to harbor the minor (alternative; ALT) SNP of *ZR3* and *CGR3*. In both genes, the SNP is correlated with changes in transcript abundance (Figs. 1D and 2D). Plants in the continental interior experience greater temperature variability. This is exemplified by the positive correlation between distance from the coast and temperature seasonality (BIO4) and between distance from the coast and annual temperature range (BIO7) for the Eurasian accessions in this study (Figs. 1E and 2E). These two climate variables also each show an inverse correlation with transcript abundance of *ZR3* and *CGR3*, with the minor (alternative) SNP associated with lower expression values in each case (Figs. 1F, G and 2F, G). This supports the hypothesis that these putative riboSNitches may play a role in regulatory responses to temperature variation.

### Experimental verification of climate-associated riboSNitches by UV melts

AT3G54826 (*ZR3*) and AT5G65810 (*CGR3*) each have a G-to-A variant. If these SNPs change the structure of the RNA, then they likely change the thermodynamic stability of the local fold as well. To experimentally test this idea, we designed model RNA oligonucleotides containing the SNPs of interest and assessed their folding stability by UV-detected melts. We chose minimal sequences of 17 nt (for Z*R3*) or 23 nt (for *CGR3*) on the basis of including the predicted local RNA structures flanking the identified SNP in both the reference (major allele) and alternative (minor allele) sequence.

**Fig. 3:**
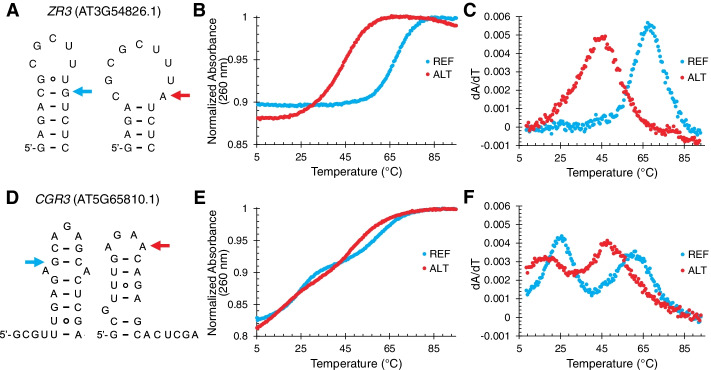
SNPs affect the thermal stability of RNA oligonucleotides as shown by UV-detected melts. **A** Predicted secondary structures of RNA oligonucleotides from AT3G54826 (*ZR3*) used in the melts. The left-hand structure is the reference, and the right-hand structure is the G-to-A variant. Colored arrows (blue for reference and red for variant) mark the sequence change. **B** Representative UV-detected thermal denaturation for the reference (blue) and variant (red) from **A**. This melt was collected at 6.5 μM of the reference and alternative oligonucleotides. **C** First-derivative plots of the data from **B**. **D** As in **A** but for AT5G65810 (*CGR3*), also with a G-to-A mutation. **E** Representative UV-detected thermal denaturation for the reference (blue) and alternative (red) from **D**. This melt was collected at 4.5 μM of the reference and alternative oligonucleotides. **F** First-derivative plots of the data from **E**. Melts of all four sequences at other concentrations are provided in Additional file 4: Fig. S1

As shown in Fig. 3A, the reference 17-nt *ZR3* was predicted to fold into a stem-loop with a six-base pair stem (five Watson-Crick pairs and a G•U wobble pair) and a five-nucleotide loop. This portion of the alternative *ZR3* sequence was also predicted to form a stem-loop (hairpin), with the same four base pairs at the base of the stem but with two fewer base pairs at the top of the stem and thus a nine-nucleotide loop. This is because the G-to-A mutation changed the penultimate base pair at the top of the stem of the reference sequence from a Watson-Crick CG base pair to a CA mismatch. Indeed, free energies, widely available at 37 °C (ΔG°_37_), for the reference and variant sequences, gave a large ΔΔG°_37_ of + 4.4 kcal/mol in going from the reference to alternative sequence, as predicted by RNAstructure [38].

We experimentally tested the stability of this pair of sequences by performing UV-detected melts using 150 mM KCl and 0.5 mM MgCl_2_, which mimic in vivo conditions for plants. As shown in Fig. 3B, the reference sequence (blue) was much more stable than the variant (red). The melting temperature (*T*_M_) of the reference sequence, revealed in the maximum in the first derivative plots (Fig. 3C.), was ~ 66 °C, while that for the variant was only ~ 44 °C. This behavior is robust to a range of RNA concentrations (Additional file 4: Fig. S1), supporting the transition being from the stem-loop rather than from a duplex. We thus calculated the thermodynamics from these melting transitions using a two-state model (state 1: stem-loop, state 2: unfolded) and obtained an experimental ΔΔG°_37_ of + 3.8 kcal/mol, in good agreement with the predicted value above of + 4.4 kcal/mol, supporting the assignment of the folds in Fig. 3A to these stem-loop structures. The melting transitions are fairly broad, but despite this, they remain well separated. The alternative sequence begins to melt at ~ 24 °C and is ~ 43% unfolded at 42 °C; on the other hand, the reference sequence is fully folded even at 42 °C (Fig. 3C). Overall, these observations support the notion that the G-to-A SNP in *ZR3* could lead to a “conditional” riboSNitch”: a SNP that manifests as a structural change only under a certain condition; here, one of heat when the temperature is elevated above ~ 25 °C.

We next turned to the AT5G65810 (*CGR3*) minimal sequences of 23 nt, which encompass the predicted local RNA structures flanking the identified SNP in both the reference and alternative allele. The reference 23 nt *CGR3* sequence was predicted to form a stem-loop with a seven-base pair stem (six Watson-Crick pairs and a G•U wobble pair penultimate to the base of the stem) containing an AA mismatch, as well as a three-nucleotide loop (Fig. 3D). The alternative *CGR3* sequence had a completely different structure. It was predicted to form a stem-loop with a six-base pair stem (five Watson-Crick pairs and a G•U wobble pair) with a 5′-strand G bulge, as well as a four-nucleotide loop. Indeed, no base pairs were shared between the 23-nt reference and alternative structures despite them varying by only a single base; as such, this SNP is a potential riboSNitch for this portion of the sequence even at low temperatures, unlike the case of *ZR3*. To accommodate the two very different structures in the reference and alternative, our set of paired reference and alternative oligonucleotides resulted in a tail of four nucleotides found on the 5′-end of the reference sequence and a tail of six nucleotides found on the 3′-end of the alternative sequence (Fig. 3D). The predicted ΔG_°37_ values for these two structures were fairly similar at − 4.0 and − 2.9 kcal/mol, again consistent with the notion that these two sequences are riboSNitches even at low temperatures and that these should melt over approximately the same temperature range.

As before, we tested the stability of this pair of sequences by UV-detected melts in in vivo-like salt conditions. As shown in Fig. 3E, F, both sequences melt over a broad temperature range of ~ 10 to 75 °C. The first (low temperature) melting transition shifted higher in *T*_M_ with RNA concentration and so is assigned primarily to melting of a duplex while the second (high temperature) transition did not shift with RNA concentration and so is assigned to melting of a hairpin (see Additional file 4: Fig. S1). The duplex melting is irrelevant at the low concentrations of RNA found in vivo; thus, we focus on the second transition. The alternative sequence melts at a lower temperature (*T*_M_ ~ 47 °C) than at the reference (*T*_M_ ~ 60 °C), but on the other hand, both melting temperatures are well above biological values. Thus, the G-to-A SNP in *CGR3* could lead to a riboSNitch throughout the standard temperature range of *Arabidopsis*, i.e., it has characteristics of a persistent or “classical” riboSNitch.

### Experimental demonstration of climate-associated riboSNitches by gel-based structure probing

**Fig. 4:**
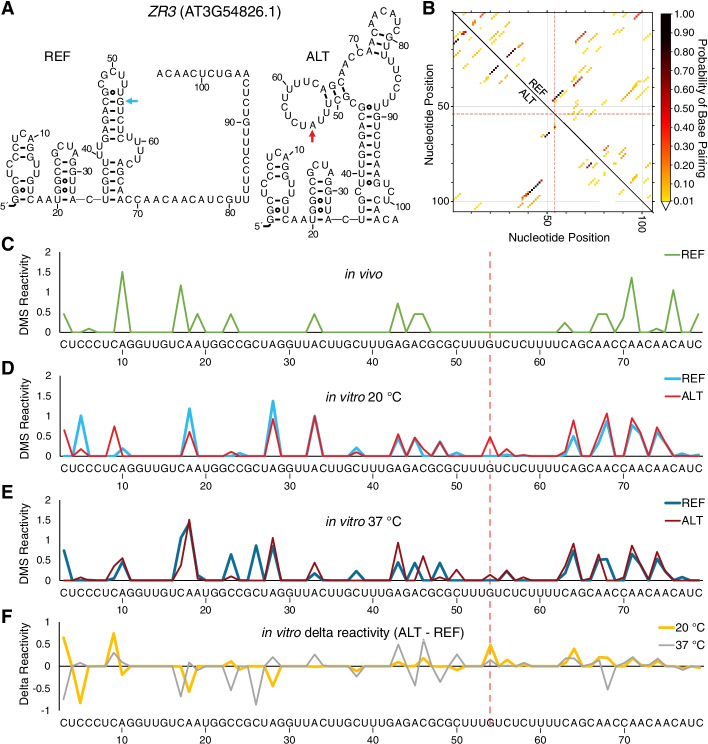
The G-to-A SNP changes the secondary structure of an RNA segment from *ZR3*. **A** Predicted secondary structures for 105-nt *ZR3* reference and alternative sequences (chr3:20310428–20310522, 20311150–20311157), including the added GG at the 5′-end added to improve T7 transcription, making the final length 105 nt. The SNP is identified by an arrow. Structures were folded using RNAstructure [38] and outputted using R2R (https://help.rc.ufl.edu/doc/R2R) [40]. **B** MutaRNA dot plot [39] displays the probability of base pairing of the reference (above the diagonal) and alternative (below the diagonal) sequences. The color scale shows the strength of base pairing probability. The position of the SNP is denoted with dashed red crosshairs. **C**–**F** DMS reactivity of a 77-nt portion of the *ZR3* RNA segment (nucleotides corresponding to the primer binding site for the in vitro probing and compressed data at the top of the gel could not be mapped) is plotted in **C** from an in vivo dataset from plants grown at 21 °C [6], as well as for two in vitro DMS experiments conducted at **D** 20 °C and **E** 37 °C. In vitro DMS reactivity was derived from two sequencing gels provided in Additional file 4: Fig. S2. **F** The delta DMS reactivity of the alternative and reference sequences at both 20 °C and 37 °C

Next, we assessed the effect of each SNP in a longer RNA context. We began with an RNA designed from *ZR3*. While in the previous experiment we melted only the minimal predicted structure encompassing the SNP, here, we designed a pair of RNAs with *ZR3* sequences 51 nt upstream and 51 nt downstream of the SNP for sequences differing only in the identity of the SNP. As indicated in Fig. 4A, the two RNAs have very different in silico predicted folds, suggesting that the SNP might indeed be a riboSNitch. We then carried out an analysis by MutaRNA [39], a web server for visualization and interpretation of mutation-induced changes in RNA structure (http://rna.informatik.uni-freiburg.de/MutaRNA/Input.jsp). This program presents probabilities of base pairing for multiple structures of a sequence and displays differences between a pair of sequences as differential heatmap-like dot plot representations. In Fig. 4B, the reference is represented above the diagonal and the alternative below, and the position of the SNP is indicated with crosshairs. The diagram presents in a single representation the set of most probable folds of each sequence; in other words, a given nucleotide can have more than one possible partner such that different copies of the same sequence can adopt different folds, each designated with a color-coded probability. Inspection of the data for nucleotides farthest from the crosshairs indicates similarities in folding. For instance, near the top left corner of Fig. 4B, the pattern of dots, representing helices in a given set of folds, are largely distributed symmetrically about the diagonal, albeit often with different shading across the diagonal, indicating different probabilities of forming the folds within the set. Notably, data closest to the crosshairs show more different folding possibilities. For instance, there are helices that appear on one side of the diagonal that are missing on the other, or the shading is extremely different between the sequences. There are some exceptions to SNP-local changes in folding, however, especially near the bottom right corner of the diagram, where some helices are missing altogether in the alternative, suggestive of potential long-range structural effects of the SNP in addition to the local ones.

We then turned to experimentally probing the effect of the SNP on the structure of these sequences. The structure of each RNA was probed with dimethyl sulfate (DMS), which covalently modifies unpaired As and Cs on the Watson-Crick face, resulting in reverse transcription DMS-induced “structure-stops,” which can be read out on sequencing gels. The structure-probing gels are provided in Additional file 4: Fig. S2, and the quantification is provided in Fig. 4C–F. The first trace (Fig. 4C) presents the in vivo DMS structure-probing results from our published studies on *Arabidopsis* [6]. There are multiple peaks along the course of this RNA, which signify regions that were unpaired and not interacting with proteins. The in vitro data at 20 °C and 37 °C are provided in Fig. 4D and E, respectively, for both the reference and alternative, with difference plots between reference and alternative at each temperature provided in Fig. 4F. Inspection of Fig. 4D for in vitro structure probing reveals nearly identical plots for the reference and alternative at 20 °C, consistent with both RNAs being in the folded baseline of the melts at this temperature (Fig. 3B). This is corroborated by the difference plot (Fig. 4F, yellow), which shows relatively minor features. The comparison of the in vitro 20 °C (Fig. 4D) to the in vivo 21 °C data (Fig. 4C) reveals numerous features in common, suggesting similar folding of at least this portion of the transcript in vitro and in vivo and supporting the relevance of our in vitro analyses. There are a few peaks that are uniquely present in vitro, suggesting that these may serve as protein binding sites in vivo. Figure 4E for in vitro structure probing at 37 °C reveals much more significant structural differences for the reference and alternative than at 20 °C, consistent with a change in structure during heat stress. In some cases, the reference sequence is more accessible to DMS, and in other cases, the reverse is true. This is again corroborated in the difference plot (Fig. 4F, gray), which shows both positive and negative features that are more significant than the 20 °C features (Fig. 4F, yellow). These differences are near the SNP (red dashed vertical line) but extend further out, especially towards the 5′-end. That the effect is not simply due to greater DMS reactivity at higher temperature is supported by flips in the algebraic signs, and not just the magnitude, of the features at the two temperatures (Fig. 4F, compare yellow and gray).

**Fig. 5:**
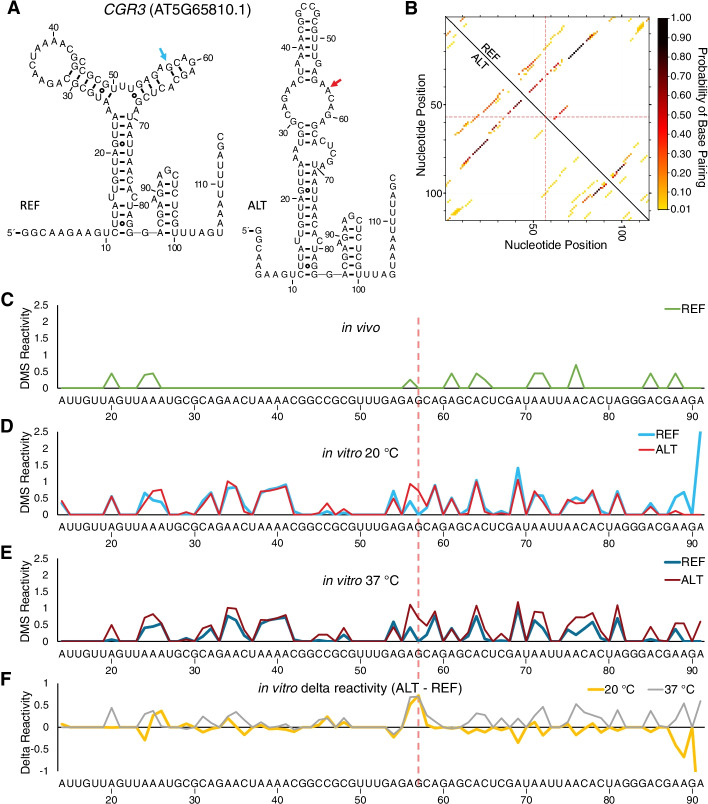
The G-to-A SNP changes the RNA secondary structure of an RNA segment from *CGR3*. **A** Predicted secondary structures of 115-nt *CGR3* reference and alternative sequences (chr5:26339678–26339792). The SNP is identified by an arrow. Structures were folded using RNAstructure and outputted using R2R (https://help.rc.ufl.edu/doc/R2R) [40]. **B** MutaRNA dot plot [39] displays the probability of base pairing of the reference (above the diagonal) and alternative (below the diagonal) sequences. The color scale on the right shows the strength of base pairing probability. The position of the SNP is denoted with the dashed red crosshairs. **C**–**F** DMS reactivity of a 78-nt-long portion of the 115-nt-long *CGR3* sequence (nucleotides corresponding to the primer binding site for the in vitro probing and compressed data at the top of the gel could not be mapped) is plotted in **C** from an in vivo dataset from plants grown at 21 °C [6], as well as for two in vitro DMS experiments conducted at **D** 20 °C and **E** 37 °C. In vitro DMS reactivity was derived from two sequencing gels provided in Additional file 4: Fig. S2. **F** The delta DMS reactivity of alternative and reference sequences at both 20 °C and 37 °C

We then carried out the same set of analyses on an RNA designed from *CGR3*. Again, we designed a pair of RNAs based on the native *CGR3* sequence, with 56 nt upstream and 58 nt downstream, differing only at the SNP. This pair of RNAs also had very different folds according to RNAstructure (Fig. 5A). Analysis by MutaRNA revealed similarity of folding for regions furthest from the SNP, with differences found mainly for the alternative having a unique set of possible pairings between the SNP and distal sequences near the 3′-end (Fig. 5B). This distal pairing is suggestive of potential long-range structural effects of the SNP. Inspection of Fig. 5D for in vitro structure probing at 20 °C revealed some variation about the SNP for the reference and alternative. Comparison of the in vitro 20 °C to the in vivo 21 °C data in Fig. 5D and C, respectively, reveals numerous features in common but also shows multiple peaks missing in vivo, suggestive of significant protein binding in vivo, albeit away from the SNP. Figure 5E for in vitro structure probing at 37 °C reveals, as for *ZR3*, much more significant structural differences between the reference and alternative than at 20 °C, again consistent with a change in structure under heat stress. In general, the alternative sequence is more accessible to DMS reactivity at elevated temperature, consistent with an earlier melting temperature (Fig. 3F). The difference plots (Fig. 5F) confirm these effects, with relatively minor differences at 20 °C and larger differences at 37 °C. The greater changes in reactivity owing to the SNP for both sets of RNAs at elevated temperature is consistent with both *ZR3* and *CGR3* having characteristics of conditional riboSNitches.

### Pan-structurome prediction of RiboSNitches

**Fig. 6:**
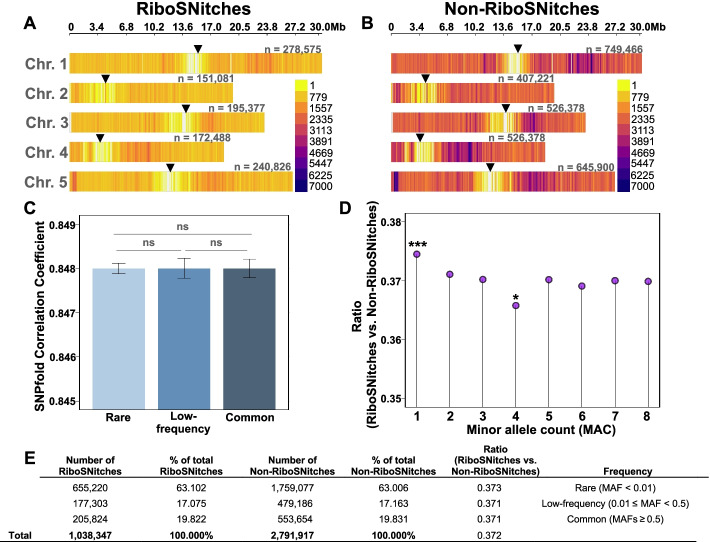
Predicted riboSNitches and non-riboSNitches in the population of 879 Eurasian accessions do not differ in their chromosomal densities and allelic frequencies. **A**, **B** The genome-wide SNV density, expressed as number of variants per 100,000 nt, for **A** 1,038,347 riboSNitches and **B** 2,791,917 non-riboSNitches. The genome-wide distribution of riboSNitches and non-riboSNitches is not significantly different (Wilcoxon *P* value > 0.05). Arrowheads depict the centromeric regions with lower SNV densities. **C** The average SNPfold correlation coefficient does not differ significantly among rare, low-frequency, and common variants (Wilcoxon *P* value > 0.05). **D** Among rare variants, at the lowest possible allele frequency (MAC = 1), the ratio of riboSNitches to non-riboSNitches is higher than the expected ratio for rare SNVs (post hoc tests following a chi-square, using the Bonferroni adjustment, *P* value < 0.001). Significant differences also occur in the opposite direction within rare variants (MAC = 4; post hoc tests following a chi-square, using the Bonferroni adjustment, *P* value < 0.05). **E** Table summarizing the frequencies and ratios of candidate riboSNitches and non-riboSNitches

We ran SNPfold on the predicted transcriptomes of the 879 Eurasian *Arabidopsis* accessions for which there is robust genome sequence and geo-referencing available. SNPs were considered candidate riboSNitches if their correlation coefficient comparing the reference and alternative structural ensembles was < 0.8 [2]. The results are summarized in Table 1, which presents the number of predicted riboSNitches/non-riboSNitches and their disposition across the transcript. Comparison of riboSNitches to non-riboSNitches suggests there is no bias in their distribution within genic regions (Table 1; Fig. 6).

**Table 1:** RiboSNitch abundance and distribution by genomic region. The results are compiled from the analysis of the transcriptomes of the 879 geo-referenced Eurasian *Arabidopsis* accessions with full genome sequence. riboSNitches were designated according to the criterion of Halvorsen et al. [2]. SNP, single nucleotide polymorphism; rbSN, riboSNitch; non-rbSN, non-riboSNitch

Gene region	SNPs	rbSN	Non-rbSN	rbSN/SNPs	rbSN/non-rbSN
All	3,830,264	1,038,347	2,791,917	0.27	0.37
Missense variants	1,003,976	280,246	723,730	0.28	0.39
Missense variant and splice region variants	15,275	4698	10,577	0.31	0.44
Synonymous variants	725,171	190,732	534,439	0.26	0.36
3′ UTR variants	401,263	108,147	293,116	0.27	0.37
Stop gained	21,954	5766	16,188	0.26	0.36
Stop gained and splice region variants	465	129	336	0.28	0.38
5′ UTR variants	246,576	60,695	185,881	0.25	0.33
5′ UTR premature start codon gain variants	36,060	9808	26,252	0.27	0.37
Intron variants	1,254,339	344,296	910,043	0.27	0.38
Splice acceptor variant and intron variants	3622	1001	2621	0.28	0.38
Splice region variants	2359	674	1685	0.29	0.40
Splice donor variant and intron variants	3581	1079	2502	0.30	0.43
Splice region variant and intron variants	100,386	27,018	73,368	0.27	0.37
Splice region variant and stop retained variants	22	7	15	0.32	0.47
Splice region variant and synonymous variants	11,434	3078	8356	0.27	0.37
Start lost	1031	281	750	0.27	0.37
Start lost and splice region variants	11	3	8	0.27	0.38
Stop lost	1277	356	921	0.28	0.39
Stop lost and splice region variants	21	9	12	0.43	0.75
Stop retained variants	1235	270	965	0.22	0.28
Initiator codon variants	202	53	149	0.26	0.36
Initiator codon variant and splice region variants	4	1	3	0.25	0.33

### T-CLIM

It has been increasingly realized that variation in transcript abundance provides significant clues regarding genes that underlie whole-organism phenotypic outcomes [41, 42]. Accordingly, we created the web tool, T-CLIM, which integrates transcriptome landscapes and environmental clines in *Arabidopsis*. T-CLIM allows users to detect variation in transcript abundance that is correlated with variation in any of 465 continuous geo-climatic parameters. The discovery of such co-variation via T-CLIM implicates the associated gene in adaptation to that climate condition. Although post-transcriptional mechanisms also control net transcript abundance as quantified in RNA-seq databases, the user can then apply GenoCLIM or CLIMGeno to test the specific hypothesis that transcript abundance variation is correlated with, and thus potentially caused by, *cis*-genetic variation, including that induced by candidate riboSNitches.

### CLIMtools V.2.0

In addition to the new T-CLIM tool, we also provide a significant expansion of our original *Arabidopsis* CLIMtools databases. CLIMtools integrates information on the local environment of genetically and phenotypically defined and distinct *Arabidopsis* accessions. Within CLIMtools, AraCLIM V2.0 collates the characteristics of the local environments of sequenced *Arabidopsis* accessions collected from their natural range [43]. We make available geo-climatic variables obtained through curation of geo-environmental data resources, including high-resolution interpolated climate data, imagery data from the NASA constellation of Earth Observing System satellites, and soil databases. We extracted information on 473 climate variables, of which eight are categorical variables. This is an important expansion (Table 2) of the information included in this resource from the 204 environmental variables previously included in AraCLIM V.1.0 [36]. Environmental data descriptors and associated statistics are compiled in Table S4 (Additional file 5), while correlations among these climate variables are provided in Figure S3 (Additional file 4).

**Table 2:** Comparison of CLIMtools V1.0 and CLIMtools V2.0. NA, not available

Tool	V1.0	V2.0
**AraCLIM**	• 204 environmental variables • 1131 accessions	• 473 (465 continuous and 8 categorical) environmental variables • 2999 accessions
**GenoCLIM**	• 204 environmental variables •Mixed model GWAS (AMM)	• 465 environmental variables • Mixed (AMM) and linear model (LM) GWAS • RiboSNitch prediction • Indices of genetic diversity (PI), neutrality, and selection (Fst, Tajima’s *D*) for climate-associated variants • Integration of transcriptome × environment information for climate-associated variants
**CLIMGeno**	• 204 environmental variables • Mixed model GWAS (AMM)	• 465 environmental variables • Mixed (AMM) and linear model (LM) GWAS • RiboSNitch prediction • Indices of genetic diversity (PI), neutrality, and selection (Fst, Tajima’s *D*) for climate-associated variants • Integration of transcriptome × environment information for climate associated variants
**T-CLIM**	NA	• 465 environmental variables • Association with transcript variation in 558 Eurasian accessions with available transcriptome data

Additionally, we expand this resource beyond the set of 1131 accessions from the 1001 Genomes Project with available information on their collection site with an additional 1868 geo-referenced accessions sequenced from Chinese, African, and Madeiran accessions [44–48], plus those accessions included in the 250K global SNP array [49] for a total of 2999 *Arabidopsis* accessions with a comprehensive description of their local environment.

GenoCLIM applies genome-wide association (GWA) methods to allow the identification of associations between natural variation in any gene of interest and the extracted environmental variables in AraCLIM. CLIMGeno conversely allows, for any geo-climatic variable of interest, the identification of genes with significant co-variation with that climate variable. In our updated version of these two tools, we maintain our focus on the same set of 879 Eurasian accessions from the 1001 Genome Project as used in CLIMtools V1.0, due to the superior genome quality of these accessions. However, GenoCLIM and CLIMGeno are quite significantly expanded due to the increase in climate variables in CLIMtools V2.0 and by the inclusion of riboSNitch predictions. Further, we provide new analyses in GenoCLIM/CLIMGeno arising from the incorporation of additional analytical methods. The results are now provided not only from a GWAS mixed-model approach that corrects for population structure [50], but also from the application of a GWAS linear model [50]. While linear models do not correct for population structure, they reduce the number of false negatives in GWAS datasets. The functionality of GenoCLIM/CLIMGeno is expanded by incorporating the association between the transcript abundance of the gene containing the SNP of interest and its correlated environmental parameter, using information retrieved from T-CLIM. In addition, we have incorporated the outcomes of several common indices of diversity and selection: PI, Fst, and Tajima’s *D*.

Table 2 summarizes the new and expanded information in CLIMtools 2.0 as compared to the original CLIMtools. We provide all of these analyses in an interactive online database (https://gramene.org/CLIMtools/arabidopsis_v2.0/), available in the Gramene portal (Gramene.org) [51] for easy access.

## Discussion

Natural populations of *Arabidopsis* exhibit hallmarks of adaptation to the local climate [52]. The interplay between climate conditions and regulatory RNA structure is an important but little-studied aspect of gene regulation. Temperature is an obvious parameter to explore, given that thermodynamically heat favors RNA unfolding while cold favors the opposite. Temperature has particular implications for RNA folding in non-homeothermic organisms, especially in the current climate change scenario. We accordingly prioritized our initial wet-bench investigations of potential plant riboSNitches on those associated with temperature variables in their native environments (Figs. 1 and 2).

As shown in Fig. 3, melt analyses on oligonucleotides containing the SNP position and flanking nucleotides demonstrated differential temperature dependence of reference vs. alternative sequences during the temperature-dependent unfolding of *ZR3*. In particular, the melt data on *ZR3* clearly illustrate temperature conditionality of the SNP’s impact. Both reference and alternative oligonucleotides are fully unfolded at high temperatures and fully folded at low temperatures. However, over an intermediate temperature range, differential temperature unfolding of the reference and alternative sequences is seen, consistent with the onset of riboSNitch behavior. Similarly, melt analyses that focus on the higher temperature melt transition assignments to the *CGR3* hairpin (Fig. 3) also demonstrate differential temperature dependencies of reference vs. alternative oligonucleotides, also consistent with characteristics of a conditional riboSNitch. While Laederach and colleagues have used the term “environmental riboSNitch” to describe a SNP that behaves differently in vitro vs. in vivo [29], we propose the term “conditional” riboSNitch for those riboSNitches showing dependence on naturally occurring abiotic conditions such as temperature, salinity, and drought [5, 6].

We next confirmed that these SNPs in *ZR3* and *CGR3* are riboSNitches by the orthogonal method of gel-based structure probing. Gel-based structure probing can provide nucleotide-specific information on longer transcripts (100–150 nt), and so we correspondingly used longer RNA sequences of ~ 100 nt (Figs. 4 and 5). Longer sequences also theoretically allow additional complexity in the folding landscape beyond that available from the oligonucleotides used in melt assays. As seen in Fig. 4F, base-pairing around the SNP region is disrupted in the alternative sequence of *ZR3* at 37 °C, but little disruption is seen at 20 °C; by contrast, for *CGR3*, structural differences are seen at both temperatures but are exaggerated at 37 °C (Fig. 5F). Because of this, *CGR3* might be a riboSNitch at low temperatures too, making it both conditional and persistent. For both *ZR3* and *CGR3* transcripts, differences in structure between the reference and the alternative sequence are localized around the region of the SNP, consistent with the local folding properties of RNA [53], although more distal effects are seen in both transcripts at 37 °C (gray traces of Figs. 4F and 5F).

We then took advantage of the information collated in CLIMtools V2.0 to assess the real-world implications of these riboSNitches. The fixation index, Fst, is an index of selection that varies between 0 and 1, wherein low values indicate a lack of differential distribution of the single nucleotide variants among populations, i.e., for plants, absence of evidence that the variant is involved in local adaptation. As shown in Fig. 1, variation in *ZR3* is associated with variation in continentality (distance from the coast), which strongly co-correlates with several temperature parameters that are drivers of selection in the Eurasian *Arabidopsis* population [54]. *ZR3* exhibits an Fst of 0.44 in our analyses. As the values for the majority of Fsts for the collection of SNPs within the 879 Eurasian accessions that we have evaluated for association with continentality and related climate variables is less than 0.3 (Additional file 4: Fig. S4), this Fst value indicates selection on *ZR3*. The riboSNitch in *ZR3* is a synonymous SNP, and it will be of particular interest to assess the impact of this SNV on the *ZR3* mRNA. This variant is associated by TWAS with changes in *ZR3* transcript abundance (*cis*-regulation) (Fig. 1), suggesting a role in mRNA production or turnover, which are known to be affected by structure [5]. We additionally evaluated the transcriptomes available from 558 accessions [37] for a potential effect of this SNV in alternative splicing, but found no differences in splice site choice between accessions harboring the reference vs. the alternative SNP (Additional file 4: Fig. S5A). Translation also can be impacted by synonymous SNPs by virtue of differences in codon usage or by structural effects [55, 56]. Both the reference and alternative codons (for leucine) at this position are at a frequency of 12.54/1000 in the *ZR3* transcripts in this population, suggesting that natural selection at this site does not reflect differential codon usage bias for reference vs. alternative codons. mRNA structural effects on translation efficiency have been strongly associated with a window from − 4 to + 37 nucleotides of the translation start [57]. The *ZR3* SNV, at position + 125, is not within this window. However, given that RNA structural effects of a riboSNitch can propagate away from its site, structural changes associated with the *ZR3* riboSNitch may still affect ZR3 protein production.

*ZR3* encodes a mitochondrial protein with homology to the Hep1 protein of *Saccharomyces cerevisiae*, and *Arabidopsis ZR3* functionally complements *hep1* yeast knockouts [58]. Yeast *Hep1* has an essential function in thermotolerance: the Hep1 protein chaperones heat shock protein mtHsp70, which itself is a chaperone that opposes misfolding of proteins under heat stress [59]. *Hep1* deletion mutants accumulate mtHsp70 aggregates and exhibit conditional lethality, dying at elevated temperatures [58, 59]. Our results suggest that it would be of great interest in future studies to compare these variant *ZR3* sequences in yeast complementation assays over a temperature cline, especially given that plant Hsps are also associated with cold tolerance [60, 61] and that *ZR3* variation is not associated with absolute temperature parameters, but rather with temperature variation (Fig. 1).

The 5′ UTR SNP in *CGR3* shows striking evidence of selection, with a very high Fst of 0.73. This result, coupled with our observation that this variant is associated with differential *CGR3* transcript abundance, adds to the growing body of evidence that non-coding SNPs in transcripts have functional impacts. While variants in 5′ UTRs could theoretically affect upstream ORFs or transcription start sites (TSS), our analyses (Additional file 4: Fig. S5B) of the transcriptomes analyzed herein produced no evidence of different TSS for *CGR3* reference vs. alternative sequences, leading us to focus on a role for RNA structure. As shown in Fig. 2, variation in *CGR3* transcript abundance is associated with variation in continentality and is also associated with variability in other thermal attributes of the geo-referenced collection sites, suggesting an expression by environment interaction that is influenced by 5′ UTR structure. CGR3 is a Golgi-localized protein with homology to pectin methyltransferases, which methylesterify pectin, a major non-cellulosic component of the plant cell wall. Knockout of *CGR3* and the homologous *CGR2* gene results in plants with decreased methyltransferase activity and reduced levels of cell wall pectin methylesterification [62, 63]. The extent of methylesterification has been shown to affect thermal acclimation. Null mutants of the pectin methylesterase *PME34* show increased susceptibility to heat stress [64] but, interestingly, acclimation to cold also has been associated with increased pectin methylesterification [65]. Our results suggest that it could be informative to assess variants of *CGR3* for their impact on the production of *CGR3* protein, *CGR3* enzymatic activity, and relative tolerance of temperature extremes.

In sum, we have identified two plant riboSNitches with strong functional implications and have confirmed each of them by two orthogonal experimental techniques. Given that Laederach and colleagues, in their evaluation of riboSNitch prediction algorithms considered just 11 human SNP pairs as “gold standard” riboSNitches [29], i.e., verified by two orthogonal wet bench methods, this first experimental verification of plant riboSNitches adds considerably to that resource.

Having demonstrated the existence of plant riboSNitches, and provided evidence that those we studied experimentally are under positive selection, we then moved on to pan-structurome-wide prediction of *Arabidopsis* riboSNitches from 879 Eurasian accessions. Out of the 3,830,287 transcriptomic SNPs evaluated computationally, 1,038,347 or ~ 27% were designated as candidate riboSNitches (Table 1). This percentage is reasonable given that Wan et al. found that 15% of SNPs are riboSNitches in an experimental comparison that involved just three human genomes and that targeted analysis of two human transcripts also revealed structural changes in ~ 15% of variants [66].

Our pan-structurome-wide analyses of 879 *Arabidopsis* transcriptomes allowed the identification of some interesting features associated with plant riboSNitches. The database of human riboSNitches, RiboSNitchDB, provides information on 24,629 eQTL-associated predicted riboSNitches plus several hundred others with experimental support [30]. In comparison, our identification of 1,038,347 predicted riboSNitches (Table 1) available in CLIMtools V2.0 provides perhaps the largest queryable dataset on riboSNitches of any organism. This large dataset allows us to evaluate riboSNitch characteristics. The genome-wide chromosomal distribution of riboSNitches vs. non-riboSNitches was not significantly different (Fig. 6A, B). The likelihood of a SNP being predicted as a riboSNitch was identical for rare, low-frequency, and common SNPs (Fig. 6C). For our global set of riboSNitches in the Eurasian accessions, the Fsts for riboSNitches vs. non-riboSNitches did not reveal differences (Wilcoxon *P* value = 0.25), i.e., there is no evidence for overall negative selection against riboSNitches in *Arabidopsis* in the set. Similarly, the distributions of allele frequencies were not significantly different, so there was no evidence of biased purifying selection on riboSNitches (Fig. 6D, E), except possibly at a minor allele count (MAC) of one (Fig. 6D). The opposite has been proposed for human riboSNitches vs. non-riboSNitches [22]. As plants, and in particular the weedy species Arabidopsis, are subject to a broad range of environmental conditions over their environmental range, and as plants do not thermo-regulate as mammals do, it is possible that a wider range of structural variants in plants could provide adaptive strategies than is the case in mammals.

It is also of interest to assess the distribution of riboSNitches among different genic regions, given the differing functional roles of the 5′ UTR, CDS, and 3′ UTR. Among our coding sequence riboSNitches, 28% were missense (non-synonymous) variants, and among non-synonymous SNPs in the CDS, there was again no difference in the selection strength (Fst) between riboSNitches and non-riboSNitches (Wilcoxon, *P* value = 0.53), possibly suggesting that alteration of protein structure, rather than RNA structure, has a greater influence on fitness in the case of non-synonymous SNPs. For synonymous SNPs, riboSNitches have a significantly lower Fst overall than non-riboSNitches in our climate databases (Wilcoxon *P* value = 0.0017). This does suggest that RNA structural differences engendered by riboSNitches can be maladaptive in coding sequences.

In further comparison with human riboSNitches, Gorodkin and colleagues [22] applied RNAsnp to 201,213 human UTR SNPs in dbSNP. They predicted that 7.5% of the SNPs in both UTR regions were structure-disrupting, whereas we predict that a significantly higher percentage of the SNPs in *Arabidopsis* UTR regions are riboSNitches (Table 1). Moreover, *Arabidopsis* showed no significant differences in Fst values of riboSNitches vs. non-riboSNitches in either the 5′ UTR (Wilcoxon, *P* = 0.26) or the 3′ UTR (Wilcoxon, *P* = 0.70). These dichotomies between plant and animal datasets may indicate divergent regulatory architectures and adaptive strategies in mammals vs. plants.

Our provision of environmentally associated riboSNitches in CLIMtools V2.0 could, in the future, have a parallel in a human variant resource that would compile variant × environment interaction to identify environmentally induced illnesses and their interaction with genotype, i.e., a G×E×riboSNitch resource. The creation of such a G×E×riboSNitch resource could be impactful not only for humans but also for their pathogens. RiboSNitches have been identified in pathogenic viruses, including HIV [11], hepatits C [67], and SARS-Cov-2 [68]. Perhaps a CLIMtools-type resource for pathogens might help to expose the mechanistic underpinnings for the seasonality of some pathogens, particularly viruses such as influenza and SARS-CoV-2, that have RNA genomes which in theory would be susceptible to temperature-induced refolding.

However, there also remain considerable and ongoing challenges to riboSNitch prediction. The very concept of the conditional riboSNitch, by definition, implies that whether or not a SNP is a riboSNitch is a function of the dynamic microenvironment of the RNA. Corley and Laederach have correspondingly suggested that riboSNitch designations at the tail ends of score distributions will be most accurate [29]. Future riboSNitch prediction pipelines would benefit from the inclusion of temperature and solution conditions that influence RNA folding as variables—as is indeed true for computational methods of RNA structure prediction in general.

Furthermore, predicting riboSNitches pan-structurome-wide is computationally intensive. Indeed, because the complexity of the partition function used in SNPfold and other algorithms scales exponentially with RNA length, we reduced the length of flanking sequence from 60 nt on each side of the SNV, as we used in our initial identification of wet bench riboSNitch candidates, to 40 nt on each side of the SNV for our pan-structurome riboSNitch predictions to make the pipeline more time-efficient. Even with 40 nt flanking sequences (81 nt total), over 1800 h of total compute time were required to complete the SNPfold analysis of the > 3.83 million SNPs in our pan-structurome, running in parallelized fashion with 20 cores on up to 9 nodes on Penn State’s supercomputing clusters. When we analyzed riboSNitch prediction for our two specific wet bench candidates, *ZR3* and *CGR3*, as a function of flanking sequence lengths, we found significant sensitivity of the prediction to flanking sequence length (Additional file 4: Fig. S6A,B). We next assessed the 616 climate-associated SNPs that comprised our initial dataset for potential wet bench analyses (Additional file 6: Table S5) as a function of flanking sequence length and found that on average, SNPs were more likely to be predicted as riboSNitches as the flanking sequence length decreased (Additional file 4: Fig. S6C). This result is consistent with the expected local structural impacts of SNPs and supports our choice of a 40-nt flanking sequence for pan-structurome-wide folding. However, it would be demanding in compute time to expand this detailed analysis of the impact of flanking sequence length on riboSNitch prediction to, e.g., all of the ~ 3.83 million SNPs in our pan-structurome, suggesting that alternative approaches are needed. One alternative would be machine learning of riboSNitch attributes as derived from wet bench datasets. Woods and Laederach [66] pioneered one such approach for 17 different RNAs. With genome-wide methods of structure-probing now available [26] and applicable to biological systems studied under different stress conditions [5, 6], machine learning approaches based on experimental attributes would seem to hold considerable promise for the identification of riboSNitches, both persistent and conditional.

In terms of predicting functional consequences, GWA analysis of riboSNitches, as for all GWA studies, is susceptible to issues of linkage disequilibrium [9], wherein the actual functional effect could be caused by a nearby linked polymorphism, rather than by the identified SNP/riboSNitch. This limitation can be partially ameliorated by TWAS analyses, as we have done here (Figs. 1 and 2), which are less susceptible to issues of linkage disequilibrium [41, 42]. RiboSNitch and GWA analyses in humans are further complicated by heterozygosity; here, *Arabidopsis* has an advantage as it is naturally inbred, avoiding the need for disambiguation of alleles within an accession.

## Conclusions

We have experimentally demonstrated the existence of plant riboSNitches and have contributed to the still small pool of robustly validated riboSNitches in any species. Our analyses introduce and validate the concept of conditional, climate-dependent riboSNitches. Our CLIMtools resource makes possible a new approach to identify and prioritize candidate functional riboSNitches through their statistical associations with environmental parameters and transcript abundance.

The potential mechanistic impacts of riboSNitches are varied yet to date remain largely unexplored. RiboSNitches can control mRNA accessibility to the spliceosome and the ribosome, as well as to polymerases and nucleases. RNA turnover is also controlled through polyadenylation/deadenylation, which are in turn controlled by RNA structure [69, 70]; as such, riboSNitches could control RNA half-life. RiboSNitches could also affect the function of ribozymes, which are well established in plants [71, 72], by affecting ribozyme folding or even altering a catalytic residue. There is increasing recognition of the importance of the epitranscriptome in the control of gene expression [73]; not only can riboSNitches change the type of RNA modification possible at the SNP itself, but the resultant structural changes could also impact the accessibility of other sites to RNA-modifying enzymes. Finally, through impacts on intra-molecular and inter-molecular interactions, riboSNitches could affect subcellular localization such as partitioning into stress granules and other non-membranous compartments, with implications for transcript turnover and translation [74]. All of these aspects remain ripe for future investigation.

In conclusion, our CLIMtools V2.0 resource integrates information on the transcriptome and the local environment for studies of natural variation in *Arabidopsis* and will foster the identification of genes and variants that confer adaptive responses to climate conditions. A fundamental understanding of the contributions of riboSNitches to stress tolerance can contribute to future biodesigns for improved climate-resilient crop germplasm.

## Materials and methods

### RiboSNitch prediction

The SNPfold program [2] was used to identify predicted riboSNitches. In this program, the reference and variant SNP-containing sequences are described using the probability of all possible base pairs. The process includes all possible structures in the ensemble. It then compares the probabilities of all possible base pairs between the two sequences and determines the extent of correlation, wherein riboSNitches will result in reduced correlation. In this way, SNPfold computes a quantitation of the structural change caused by a natural variant. Herein, SNPs were considered riboSNitches if their correlation coefficient comparing the reference and variant sequences was < 0.8 [2].

SNPfold was applied to the 3,830,264 natural variants of protein coding genes (including introns) of the 10,293,854 SNPs within the 879 Eurasian *Arabidopsis* accessions studied here. These accessions also formed the basis of CLIMtools V1.0 and were chosen from the original 1001 sequenced genomes [43] as those that were unambigiously geo-referenced, excluding North American and British accessions that were recently (anthropogenically) dispersed and thus not yet subject to sufficient selection [43]. Each SNP in these transcriptomes was placed in the context of its surrounding 80 nt (40 nt upstream and 40 nt downstream) and passed through the SNPfold program to determine the impact of the mutation on the local RNA structure. SNPs that were located less than 40 nt from either end of a transcript (approximately 3% of transcriptomic SNPs) were excluded (designated as NC in CLIMtools V 2.0). The default parameters of SNPfold were used, in which two transcripts of equal length are compared. For the CLIMtools site, correlation coefficients are provided for all SNPs within protein-coding genes, unless the SNP was located < 40 nt from a transcript end, in which case the correlation coefficient is indicated as “not calculated.”

### Identification of riboSNitch candidates for experimental analysis

We selected a subset of 48 temperature-related environmental variables (Additional file 1: Table S1) from which we identified those SNPs with a GWAS score ≥ 4 that were in a gene that was identified as also associated with the same environmental variables through climate TWAS using a threshold based on its correlation coefficient (|*r*_*s*_| ≥ 0.3). We obtained 123 SNPs that uniquely met these conditions (Additional file 2: Table S2). Using SNPfold, we considered SNPs as candidate riboSNitches if the correlation coefficient comparing the reference and alternative sequences was < 0.8. We obtained a final list of 13 SNVs out of these 123 SNPs, within six different genes, that were predicted to be riboSNitches (Additional file 3: Table S3). As an initial screen, we used RNAstructure [38] to computationally fold transcripts of these genes containing either the reference or the alternative SNP at each of the 13 identified sites. AT3G54826 (*ZR3*) and AT5G65810 (*CGR3*) in particular showed local structural differences, as expected for riboSNitches [9], and we focused our wet bench investigations on these two mRNAs.

### Assessment of candidate riboSNitches by UV-detected thermal denaturation

The RNA oligonucleotides (see Additional file 7: Table S6) used in this experiment are 17–23 nt in length and were designed to encompass local RNA structure surrounding the identified SNP, as predicted at 37 °C using the RNAstructure software package [38]. RNA oligonucleotides were obtained from Integrated DNA Technologies (IDT) with standard desalting conditions and resuspended in DEPC-treated water at a concentration of ~ 250 μM. Ultra-0.5 mL centrifugal filters (Amicon) with a molecular cutoff of 3 kDa were used to remove any bulk sodium ions and to concentrate the RNA sample. Samples were centrifuged in a Sorvall Legend Micro 17R centrifuge (Thermo Scientific) for 10 min at 4 °C at 17,000*×g* five consecutive times. Between each spin cycle, 400 μL water was added to the approximately 100 μL of sample remaining in the column. After the final spin, the 100 μL was recovered by spinning the column upside-down at 3000*×g* for 1 min.

UV-detected thermal denaturations, or “melts,” were performed on an Olis refurbished HP 8425 diode array spectrophotometer. Melts were performed at three different RNA concentrations. Concentrations for the *ZR3* REF and ALT ranged from 1.0 to 6.5 μM, and for the *CGR3* REF and ALT ranged from 1.5 to 14 μM. These concentrations were chosen to evaluate the possible presence of intermolecular species, and reproducibility. Prior to the melts, samples were denatured at 95 °C for 2 min. in 10 mM HEPES buffer (pH 7.2) containing 150 mM KCl. After cooling to room temperature, 0.5 mM MgCl_2_ was added, and the samples were incubated at 55 °C for 3 min. Samples were again cooled to room temperature and then pipetted into 0.1 or 1 cm pathlength quartz cuvettes, slowly filling from the bottom to avoid introducing bubbles. Cuvettes were then placed in the spectrophotometer, which had been prechilled to 5 °C. RNA absorbance was monitored from 5 to 95 °C over a wavelength range of 200 to 600 nm with a data point acquired approximately every 0.5°. N_2_ gas was passed around the cuvettes from 5 °C to approximately 40 °C to minimize condensation build-up outside of the cuvettes, which would have interfered with accurate absorbance readings. A buffer reading was taken at each temperature and wavelength. We found that 260 nm gave the optimal signal. The absorbance values were buffer-subtracted and further normalized by dividing each value by the average of the five maximum absorbance values obtained for each melt. In addition, the first derivative of the normalized 260 nm absorbance values was taken, and the resultant data smoothed with 11 point moving averages.

### Assessment of candidate riboSNitches by gel-based structure probing

Gel-based structure probing can report on structural characteristics of sequences. Four gBlock gene fragments containing 51 to 58 nt of upstream and downstream sequence flanking the reference and alternative SNP of AT3G54826 (*ZR3*) and AT5G65810 (*CGR3*) were ordered from IDT. The sequences contained the T7 polymerase binding site and an additional upstream 6 nucleotides found to promote transcription. For AT3G54826 (*ZR3*), a GG sequence was added downstream of the T7 binding site to improve T7 transcription efficiency; for AT5G65810 (*CGR3*), two Gs were available from the nearby sequence. Additional file 7: Table S6 provides the gBlock sequences. Sequence lengths were slightly different between *ZR3* and *CGR3* in order to keep the SNP centered in the transcript. gBlocks were resuspended in 1X TE buffer to a concentration of 10 ng/μL per the manufacturer’s (IDT) instructions and PCR amplified using Q5 High-Fidelity DNA Polymerase (NEB) to avoid the incorporation of additional mismatches during amplification. PCR reactions (200 μL) were performed containing 40 ng gBlock template, 1 μM forward and reverse primers (see Additional file 7: Table S6 for sequences), 800 μM dNTPs, 1X Q5 reaction buffer, and 0.02 U/μL Q5 DNA polymerase. The PCR cycle consisted of an initial denaturation at 98 °C for 2 min, 10 cycles of a 98 °C denaturation for 10 s, 60 °C annealing for 30 s, a 72 °C extension for 20 s, and a final extension at 72 °C for 2 min. The PCR products were then purified using the E.Z.N.A Cycle Pure Kit (Omega Bio-tek) per the kit’s instructions and eluted in 50 μL of elution buffer.

Resultant DNA was then used in a T7 transcription to produce in vitro T7 transcribed RNA. The template DNA was denatured at 90 °C for 2 min then allowed to cool to room temperature before adding transcription buffer (final concentration in reaction: 40 mM Tris pH 8.0, 25 mM MgCl_2_, 2 mM DTT, and 1 mM spermidine), NTPs (4 mM) and additional GTP to a final concentration of 8 mM GTP, in-house prepared T7 RNA polymerase solution (50% glycerol, 50 mM NaCl, 5 mM DTT, 0.5 mM EDTA, 0.05% Triton X-100), which was added at 2 μL per 100 μL reaction, and water. The transcription reaction proceeded at 37 °C for 4 h and was quenched by the addition of an equal volume of 2X formamide EDTA loading buffer (94% formamide, 1X TBE, 0.25% bromophenol blue, and 20 mM EDTA). Transcription products were gel-purified on a 1-mm-thick denaturing PAGE gel containing 8.3 M urea and 10% acrylamide; prior to loading the samples were denatured at 95 °C for 3 min. T7 transcription products were visualized in the gel using UV-shadowing and the bands were cut out. The excised gel fragments were crushed, weighed, and incubated overnight in a rotator at 4 °C in 2× the weight by volume of TEN_250_ buffer (10 mM Tris pH 7.5, 1 mM EDTA, 250 mM NaCl). Next, the gel fragments were pelleted by centrifugation at 3000*×g* for 3 min. The resulting supernatant containing the in vitro transcribed RNA was then ethanol precipitated by adding 2× the volume of 100% ethanol, 1 μL GlycoBlue (ThermoFisher), and then incubating at − 80 °C for 2 h. The precipitated RNA was pelleted by centrifugation at 4 °C and 10,000*×g* for 30 min. The supernatant was decanted and the pellet allowed to dry before eluting in 500 μL water.

 Resultant RNA (1 μg) was then subjected to in vitro DMS probing at 20 °C or 37 °C. Samples were prepared by initially denaturing the RNA at 65 °C for 90 s, cooling on ice for 90 s, and allowing equilibration to room temperature for 5 min. RiboSNitch reaction buffer (final concentration in reaction: 10 mM HEPES, pH 7.2, 150 mM KCl, 0.5 mM MgCl_2_) was added, and samples were equilibrated at either 20 °C or 37 °C for 15 min before addition of DMS (diluted in methanol) to a final concentration of 75 mM or addition of an equal volume of methanol to minus DMS (control) samples. All DMS work was done in a hood, with proper personal protective equipment. In vitro DMS reactions were allowed to proceed at either 20 or 37 °C for 3 min. and were quenched with 2-fold excess DTT. Samples were then ethanol precipitated by adding a 2× volume of ethanol, 300 mM sodium acetate, and GlycoBlue, followed by incubation at − 80 °C for 1 h. RNA was pelleted by centrifugation at 4 °C, washed 2× with ice cold 70% ethanol, and allowed to dry before eluting in water.

For each gene, a DNA gene-specific primer (sequences provided in Additional file 7: Table S6) was radiolabeled with γ-^32^P-ATP. For each minus and plus DMS sample, 200 ng of the T7 transcript was resuspended in 6 μL DEPC-treated water and combined with 0.5 μL of the gene-specific radiolabeled primer (final concentration 200,000 cpm) and heated to 75 °C for 3 min. Samples were first pre-cooled to 35 °C and then 2 μL of reverse transcription reaction buffer and 1 μL dNTPs were added to yield final concentrations of 20 mM Tris (pH 8.3), 1 mM DTT, 8 mM MgCl_2_, and 1 mM of each dNTP, and the reaction was incubated at 35 °C. The reaction was then pre-heated to 55 °C for 1 min, and 0.5 μL of SuperScriptIII (Invitrogen) reverse transcriptase (100 U total) was added. Reverse transcription was allowed to proceed for 30 min at 55 °C. The reaction was terminated and the remaining RNA hydrolyzed by heating to 95 °C and addition of NaOH to a final concentration of 100 mM. The reactions were cooled to 4 °C prior to the addition of an equal volume of 2X formamide loading buffer (93% formamide, 1X TBE, 0.25% bromophenol blue, 1% xylene cyanol, and 20 mM EDTA). Sequencing lanes were prepared simultaneously for A, C, G, and T for both AT3G54816 (*ZR3*) and AT5G65810 (*CGR3*) sequences, by incorporating the corresponding ddNTPs to a final concentration of 1 mM in addition to 0.5 mM dNTPs and 1X transcription reaction buffer.

Reactions were visualized on an 8.3 M urea 10% acrylamide sequencing gel in which 3.5 μL of each dideoxy or sample reaction was denatured at 95 °C for 2 min. Samples were immediately loaded onto a sequencing gel pre-run at 80W. Samples were then fractionated at 80 W for approximately 2 h or until the bromophenol blue band reached the bottom of the glass plates.

Gel images were obtained using a Typhoon Phosphorimager 9410 and analyzed using Semi-Automated Footprinting Analysis (SAFA) [75]. First, each lane was normalized for total band intensity, i.e., for total counts, in order to account for any loading differences across lanes. Nucleotide identities were assigned using the sequencing lanes. Since DMS reactivity on the Watson-Crick face is specific for A and C, G and U nucleotides were excluded from downstream analysis. Differences in A and C band intensities (+DMS minus -DMS) were calculated. Normalized DMS reactivity was then calculated using the standard 2%/8% rule [76]. The top 2% of DMS reactivities per sample were initially excluded as outliers. The next 8% of DMS reactivities were averaged, and all A and C reactivities (including those of outliers) were divided by this average to obtain the DMS reactivity values shown in Figs. 4 and 5.

In vivo DMS reactivity values for the AT3G54816 (*ZR3*) and AT5G65810 (*CGR3*) mRNAs were extracted from the data of Tack et al. [6] using StructureFold2 [77]. The in vivo data were obtained on the Col-0 accession, which harbors the major (reference) allele of both *ZR3* and *CGR3*.

### AraCLIM

Climate variables were extracted as previously described [36]. The complete lists of environmental variables and summary statistics are provided in Additional file 5: Table S4.

### T-CLIM

Transcriptome-wide association (TWA) analysis of correlations between transcript abundance and each of the more than 400 environmental variables in this study was performed using the set of 558 accessions within the set of 879 Eurasian accessions that had information on transcript abundance from rosette leaves available from a previous study [37] (GEO dataset with accession number GSE80744 and SRA study SRP074107). Spearman’s rank correlation coefficients between individual climate variables and individual transcript abundance values were calculated using the correlation function of the Hmisc package (https://cran.r-project.org/web/packages/Hmisc/index.html). The stronger the association between climate and transcript variation, the closer the Pearson correlation coefficient, *r*_*s*_, will be to either + 1 or − 1. For the evaluation of our candidates to select predicted riboSNitches for experimental work, we imposed a threshold based on this correlation coefficient: |*r*_*s*_| ≥ 0.3.

### GWAS

For the calculations of genotype × environmental associations, we used a GWAS approach to identify polymorphisms in protein-coding genes (including introns) and their corresponding 1 kb promoter region that are associated with each of the 465 numerical environmental parameters included in this study. The online tool GWAPP (http://gwas.gmi.oeaw.ac.at/) was employed using an accelerated mixed model (AMM) [50]. The AMM addresses confounding effects of population stratification, family structure, and cryptic relatedness [78]. Because AMM, similar to other alternative methods designed to correct for these constraints, presents at the same time an issue with the introduction of type 2 error (false negatives), we also included information derived from a linear regression model that does not correct for population structure, also obtained using GWAPP [50], to complement our AMM analysis. We focused on SNPs within transcriptional units, including introns and including the untranslated regions at the 5′ and 3′ ends as well as 1-kb promoter regions upstream of the most distal transcription start site. We utilized the gene model annotation from the TAIR10.52 genome release that we obtained from Ensembl (http://ftp.ensemblgenomes.org/pub/plants/release-52/). We used the “consensus transcript” as the variant with the longest transcript, and predicted the effect of each individual SNP within protein-coding genes using SnpEff [79] after incorporating the annotation corresponding to TAIR10.52. We then filtered out rare variants to focus the candidate associations from GWAS that are provided in CLIMtools on common variants with MAFs ≥ 0.05 (5%). Common variants are more likely to be adaptive than rare variants, which are more likely to be undergoing purifying selection and/or arise from sequencing errors.

### Calculations of indices of genetic diversity, neutrality, and selection

We downloaded the multi-VCF for the sequenced accessions included within the 1001 Genomes Project (https://1001genomes.org/data/GMI-MPI/releases/v3.1/). We used VCFtools [80] to subset the corresponding 879 Eurasian accessions on which we focus our environmental GWAS and TWAS analysis. This step was necessary to relate this analysis with the climate associations that we conducted. We then used SnpEff and SnpSift [79] to annotate and predict the effect of every SNP, and to filter out transposons and intergenic regions. After removing indels, we filtered the resulting dataset based on missing sequencing data per site in the population of 25%, minimum quality of 40, and MAF of 5%. Genomic signatures of selection were examined in the resultant dataset by calculating the fixation index, Fst (Wright 1965; Weir and Cockerham 1984), using VCFtools, based on a per-site basis and the genetic group assigned by the 1001 Genomes Project. We also used VCFtools to calculate nucleotide diversity (*π*) and Tajima’s *D* using a sliding window of 1 kb [80].

## Supplementary Information

**Additional file 1: Table S1**. Subset of environmental variables selected to explore candidates for melts.

**Additional file 2: Table S2**. Overlapping associations retrieved from GWAS and TWAS on subset of climates selected to explore candidates for melts.

**Additional file 3: Table S3**. Final list of candidates for melts.

**Additional file 4.** This file contains Figures S1-S6.

**Additional file 5: Table S4**. Environmental data descriptors and summary statistics.

**Additional file 6: Table S5**. List of climate-associated SNVs used for riboSNitch prediction as a function of flanking sequence length (see Additional file 4: Fig. S6).

**Additional file 7: Table S6**. Oligonucleotide and primer sequences.

**Additional file 8.** Review history.

## Data Availability

CLIMtools V2.0 is web-accessible (https://gramene.org/CLIMtools/arabidopsis_v2.0/), and the latest version of its code and data are publicly available (https://github.com/CLIMtools) under the Apache 2.0 open-source license [81]. The results from GWAS and TWAS analyses, genome-wide riboSNitch predictions, as well as code and data for CLIMtools V2.0 have been uploaded to Dryad and Zenodo 10.5061/dryad.mw6m905zj [82].
