# Supplementary material for: Experimental demonstration and pan-structurome prediction of climate-associated riboSNitches in Arabidopsis
Source: Genome Biol. 2022 Apr 19;23:101. doi: 10.1186/s13059-022-02656-4 (PMC9017077; doi:10.1186/s13059-022-02656-4)
Supplement: Supplementary file 4 — Additional file 4. This file contains Figures S1-S6. [file 13059_2022_2656_MOESM4_ESM.pdf]

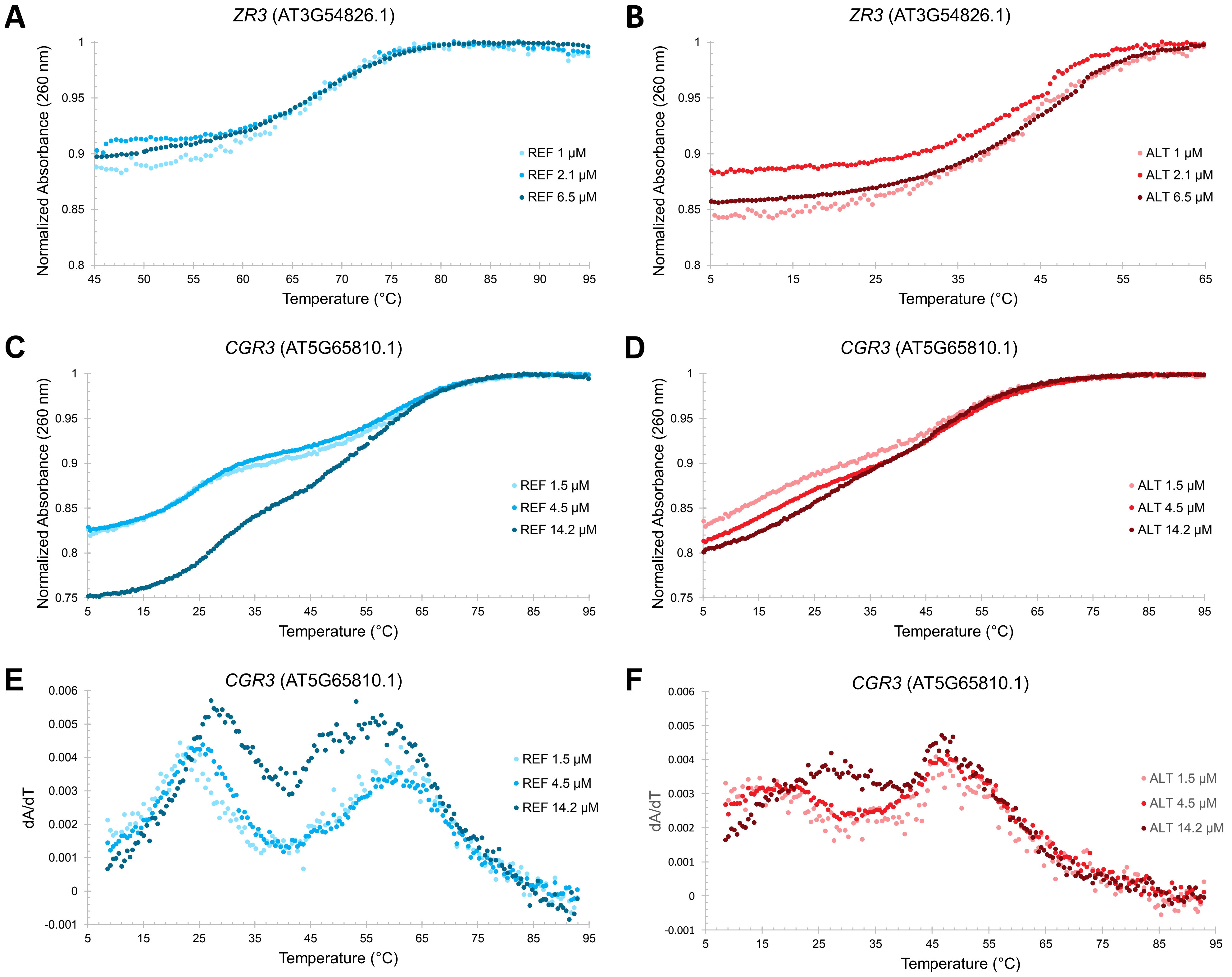

Figure S1. Assessment of concentration dependence of UV-detected melts. Overlay of representative melts of **(A)** *ZR3* reference sequence and **(B)** *ZR3* alternative sequence indicates lack of concentration dependence. The concentrations of RNA used are 1, 2.1, and 6.5 μM. Representative melts of **(C)** *CGR3* reference sequence and **(D)** *CGR3* alternative sequence. The concentrations of RNA used are 1.5, 4.5, and 14.2 μM RNA. (**E** and **F**) First derivative plots of the data in panels C and D, respectively. Observed superimposition of the first derivative maxima for the second peak support a lack of concentration dependence for the second melting transition.

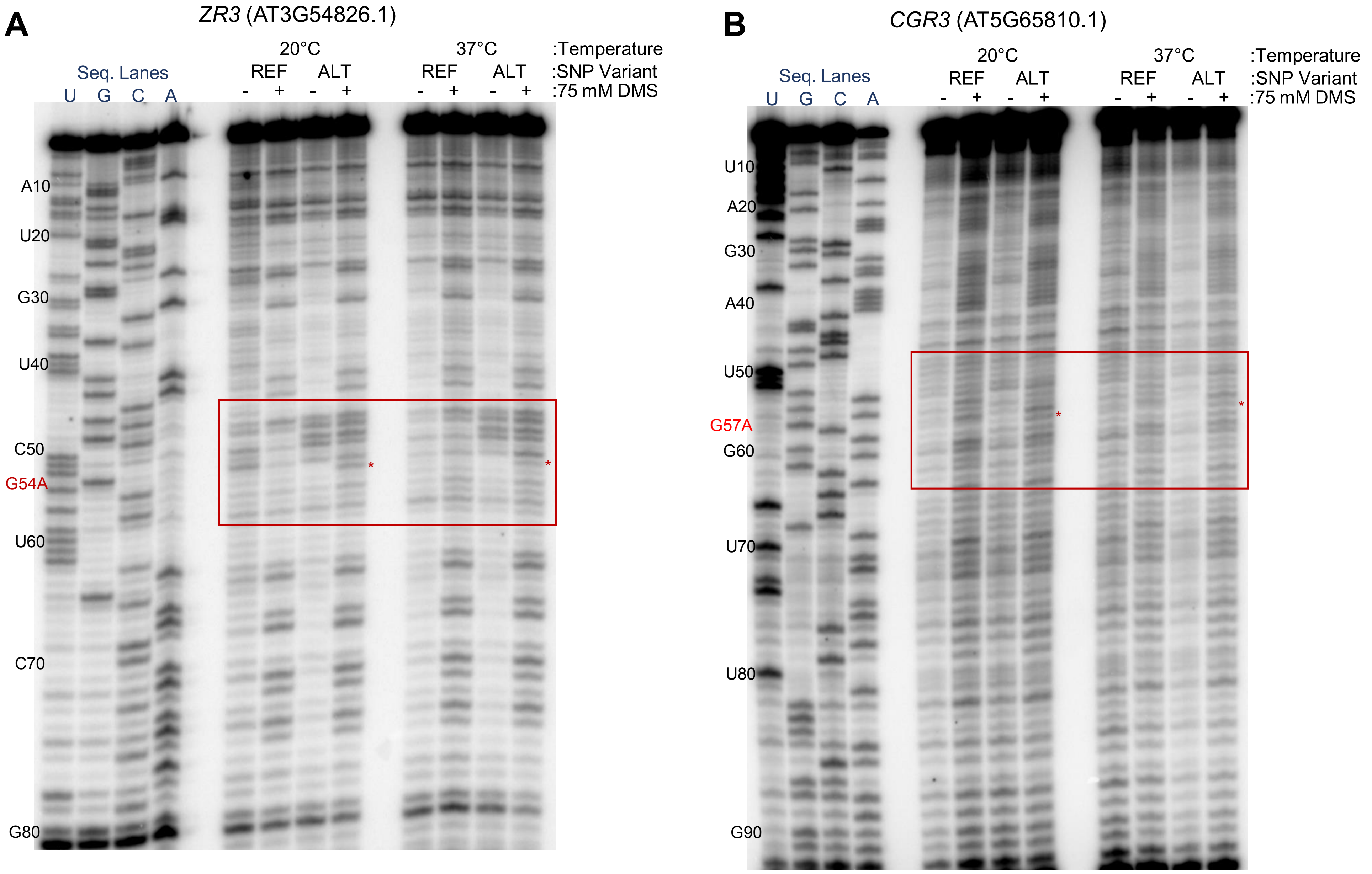

Figure S2. ***In vitro* DMS structure probing of transcribed RNAs for AT3G54826 (*ZR3*) and AT5G65810 (*CGR3*), analyzed by denaturing PAGE gels.** Sequencing gels for **(A)** *ZR3* and **(B)** *CGR3* show sequencing lanes and control and DMS reactions at 20 °C and 37 °C. The SNP nucleotides for *ZR3* and *CGR3*, G54A and G57A, respectively, are indicated in red with a nucleotide number label (on the left) and asterisk (in the gel). Areas of significant modification flanking the SNP are highlighted by a red box. The 21 and 23 3' most nucleotides for *ZR3* and *CGR3*, respectively, are not shown because the reverse transcription primer binds to them. The 13 5' most nucleotides of *CGR3* are excluded due to compression at the top of the gel.

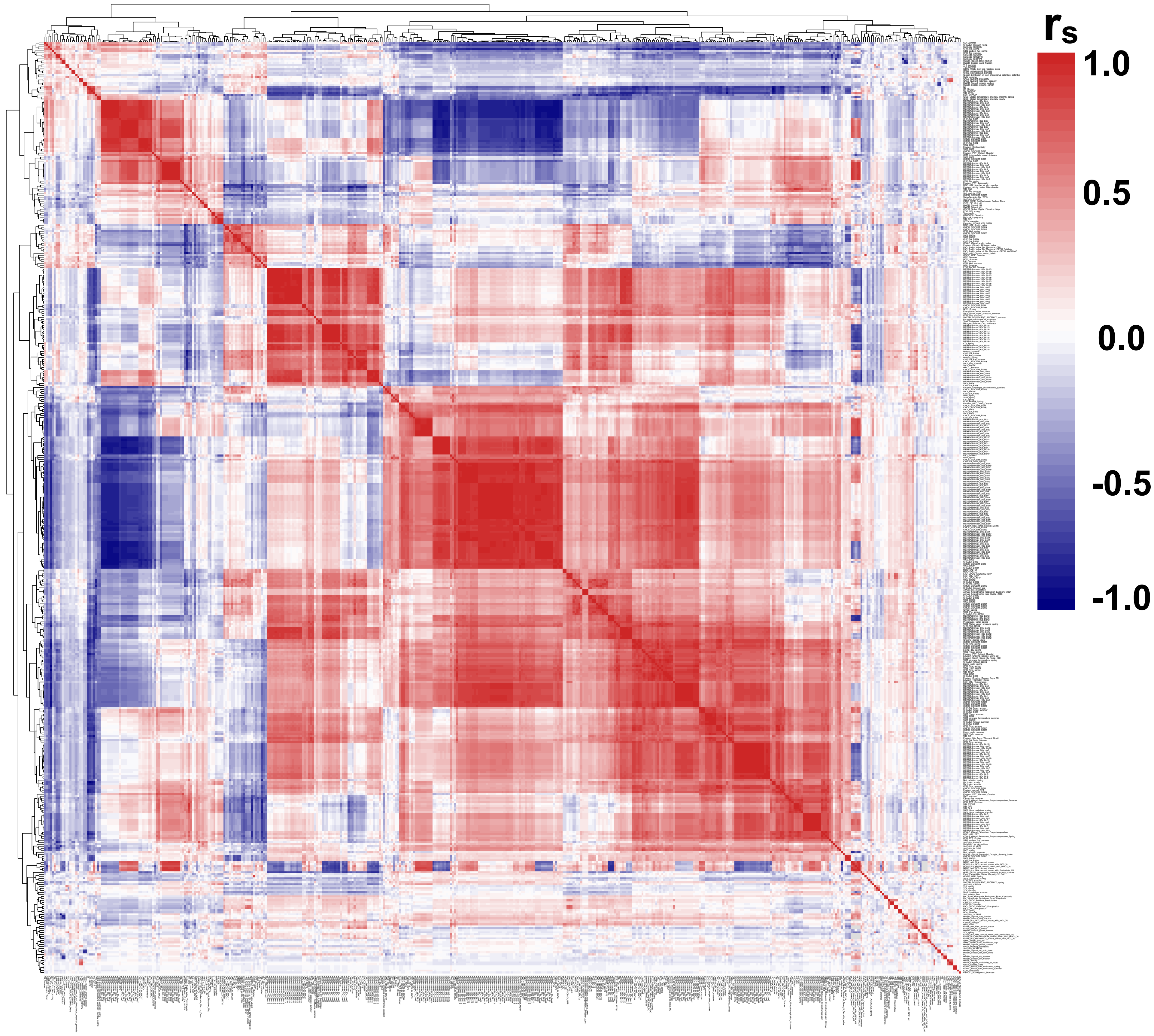

Figure S3. Correlations among the environmental variables presented in this study for the set of 465 environmental variables with continuous values. The environmental variables are ordered by hierarchical clustering. White indicates a lack of correlation, with a continuous color scale that ranges from dark blue (for strong positive correlation) to dark red (for strong negative correlation).

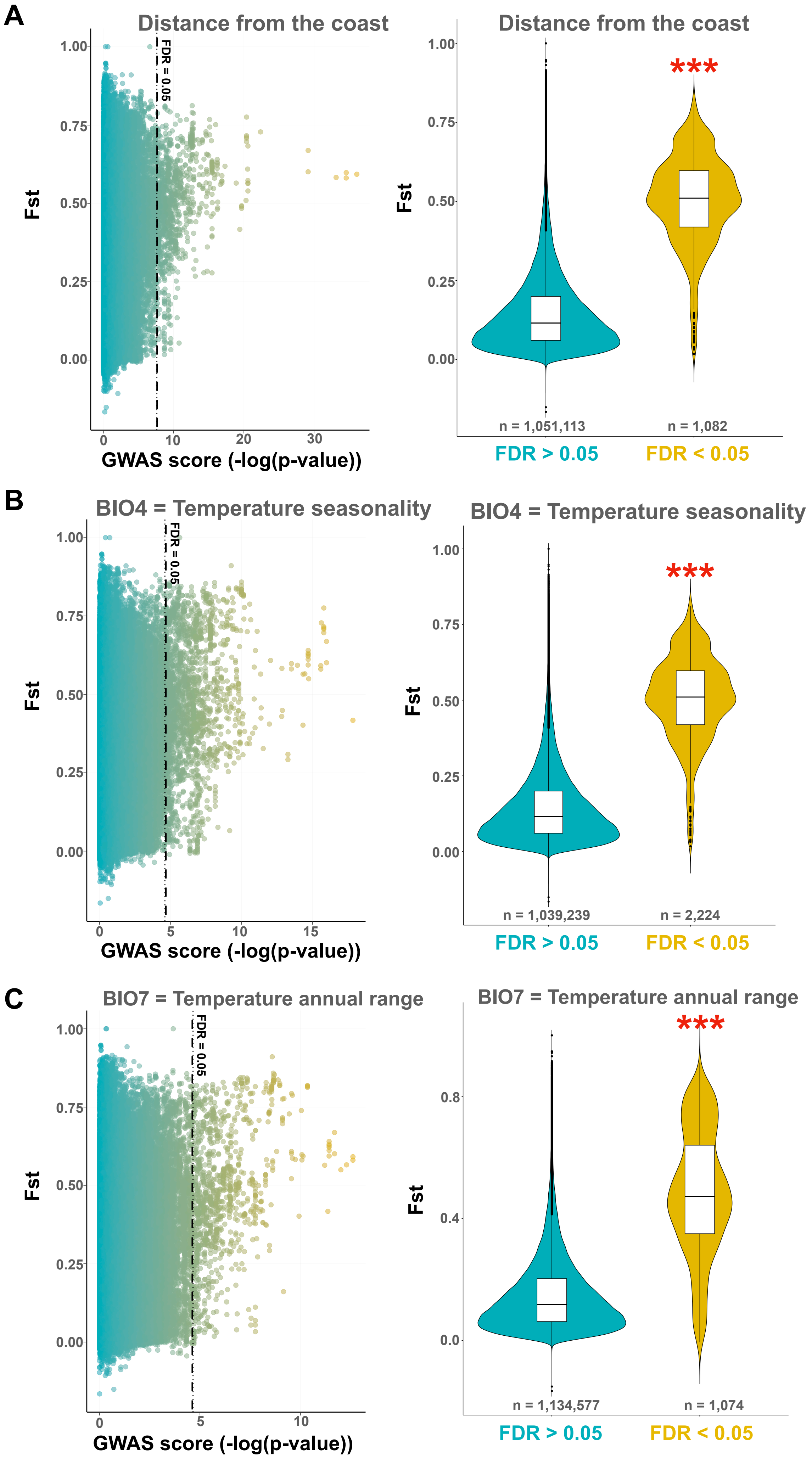

Figure S4. Distance from the coast and the associated temperature variability parameters are important drivers of selection in Eurasian Arabidopsis populations. A. Distance from the coast in the study population imposes a significant selective effect in Arabidopsis. The y-axis in both panels refers to the index of population fixation (Fst), which varies between 0 and 1, with greater values more significant for population differentiation and selection. The left panel shows how individual SNPs relate to their GWAS score resulting from applying a mixed model that ameliorates confounding effects derived from population structure. Datapoints depict individual SNPs, and the color ranging from blue to yellow illustrates the score of the association of that SNP with distance from the coast. The vertical dashed line delimits the threshold based on an FDR = 0.05 to determine a significant association between an individual SNP and distance from the coast. This is supported by the right-hand plot, which illustrates the distribution of Fst values of SNPs that are not significantly associated with this geo-environmental parameter (blue violin plot) relative to those that are significantly associated (yellow violin plot). The significant difference in both distributions (Wilcoxon, $p < 0.001$) depicts the increased population differentiation and evidence for selection in the SNPs associated with distance from the coast. Similar conclusions hold for climate parameters associated with continentality, such as the increase in temperature seasonality (BIO4; **B**) and annual temperature range (BIO7; **C**).

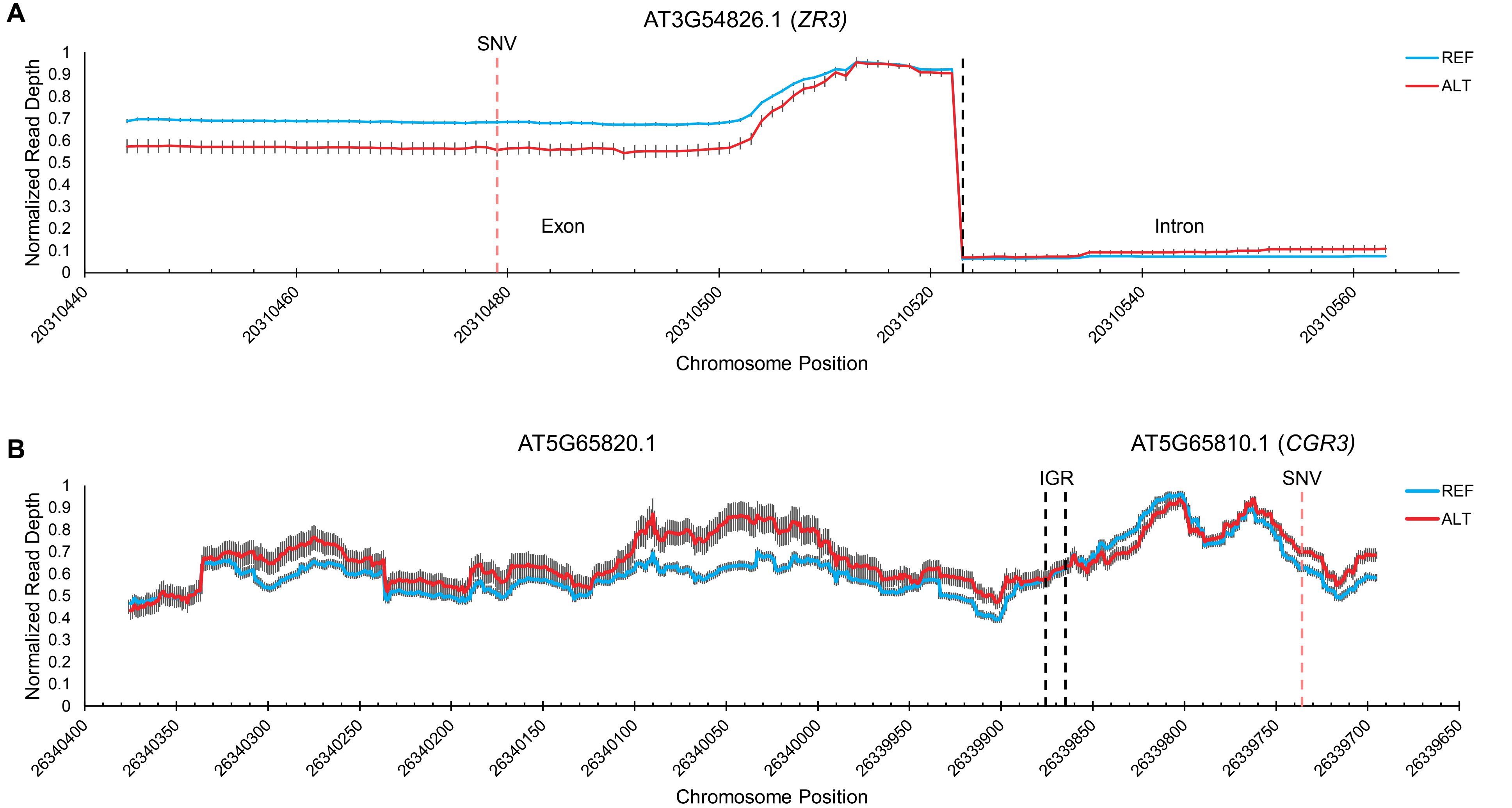

Figure S5. The presence of the alternative SNP does not affect the location of the first intron splice junction in *ZR3* or introduce alternative transcription start sites in *CGR3* in comparison to the reference SNP. Single end 1X100 Illumina HiSeq 2500 sequencing data for 558 Arabidopsis accessions [37] were downloaded from NCBI BioProject PRJNA319904 using fastq-dump (NCBI SRA-Toolkit). Read information from multiple Illumina sequencing runs per accession were concatenated into a single fastq file before continuing. Sequencing reads were trimmed for Illumina TruSeq adaptor sequences using cutadapt [1]. Trimmed fastq files were then mapped to the Arabidopsis release 52 genome assembly, downloaded from Ensembl, using either bowtie2 [2] with parameters "-p -a --no-unal", or STAR aligner [3] with parameters "--runThreadN 7 --outSAMtype BAM SortedByCoordinate", for mapping to *CGR3* and *ZR3*, respectively. Sorted mapped bam files of accessions containing the reference and alternative SNP were then assessed separately for read depth at regions of interest: the annotated *ZR3* splice junction and surrounding sequence containing the SNV location (chr3:20310444-20310563); and a portion of the *CGR3* 5'UTR containing the SNV in addition to the entire 11 bp intergenic region and the 3'-most 500 bp of upstream gene AT5G65820.1 (chr5:26339696-26340375). Read depth was normalized by dividing by the maximum read depth value in the evaluated regions of either *ZR3* or *CGR3*, respectively. The average normalized read depth across all reference (blue) and alternative (red) accessions was plotted for **(A)** *ZR3* and **(B)** *CGR3* with black standard error bars at each chromosomal location in the identified regions of interest. The dashed red line denotes the SNV location. The black dashed line in panel A denotes the start of the intron and the black dashed lines in panel B denote the borders of the intergenic region (IGR). No difference in read depth pattern in these regions of *ZR3* and *CGR3* were noted between the accessions containing the reference and the alternative SNP.

1. Martin M: **Cutadapt removes adapter sequences from high-throughput sequencing reads.** *EMBnetjournal* 2011, **17:**10-12.
2. Langmead B, Salzberg SL: **Fast gapped-read alignment with Bowtie 2.** *Nat Methods* 2012, **9:**357-359.
3. Dobin A, Davis CA, Schlesinger F, Drenkow J, Zaleski C, Jha S, Batut P, Chaisson M, Gingeras TR: **STAR: ultrafast universal RNA-seq aligner.** *Bioinformatics* 2013, **29:**15-21.

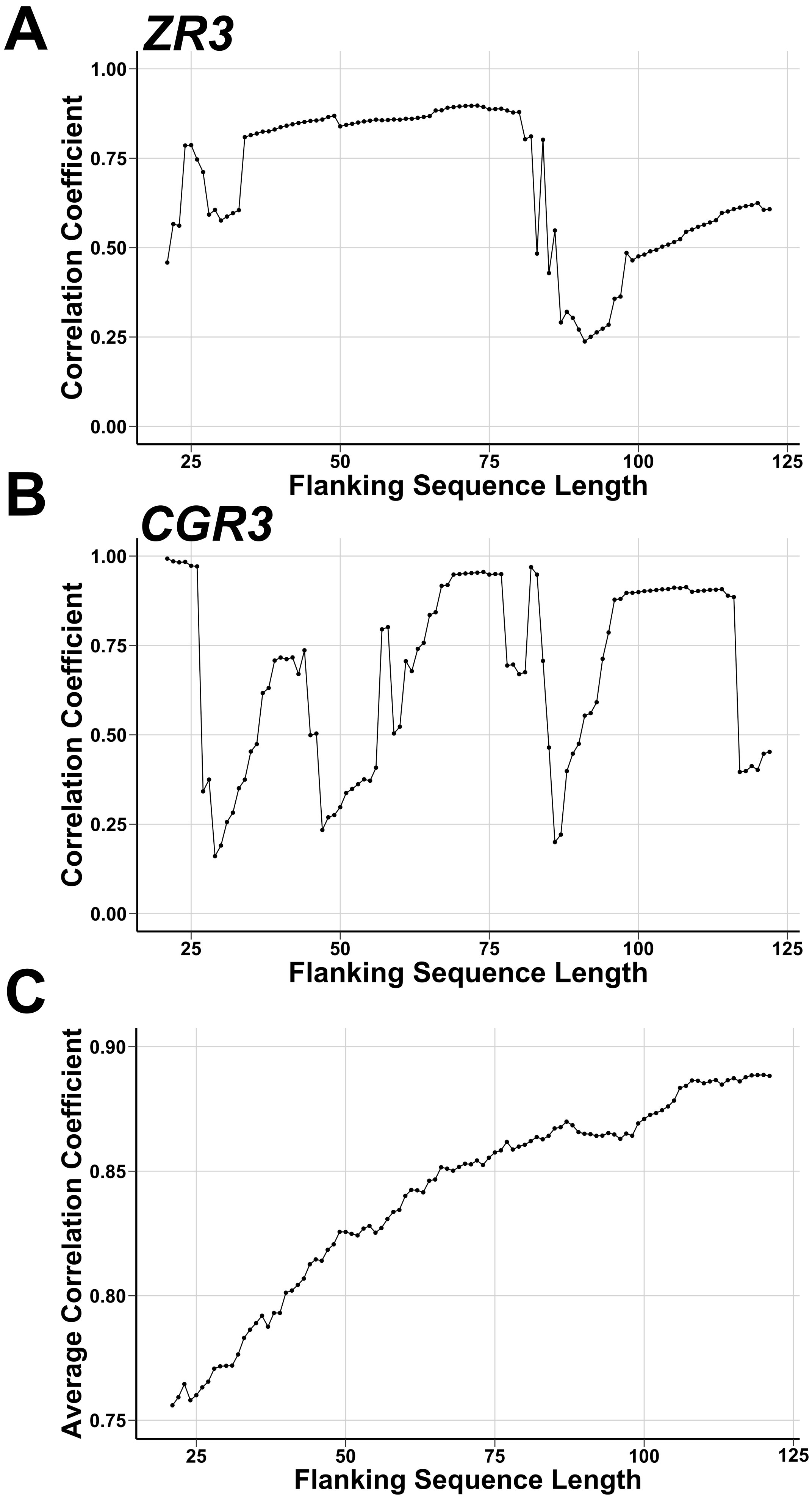

Figure S6. RiboSNitch prediction as a function of flanking sequence length. RiboSNitches were predicted using SNPfold for a range of sequence lengths of 10 nt to 60 nt flanking the centrally-located SNP (21 to 121 nt total length), with the flanking sequence length incremented one nucleotide at a time; e.g. if P symbolizes the SNP, then P, nP, nPn, nnPn, etc. Smaller values of correlation coefficients indicate more divergent structural ensembles between the reference and alternative sequence, i.e. greater probability of a riboSNitch. RiboSNitch prediction as a function of flanking sequence length for the experimentally evaluated SNP in **(A)** *ZR3* and **(B)** *CGR3*. **(C)** Average correlation coefficient as a function of length for the 616 SNP pairs initially identified as candidate climate-associated SNPs for wet bench analyses.
