# Supplementary material for: Experimental demonstration and pan-structurome prediction of climate-associated riboSNitches in Arabidopsis
Source: Genome Biol. 2022 Apr 19;23:101. doi: 10.1186/s13059-022-02656-4 (PMC9017077; doi:10.1186/s13059-022-02656-4)
Supplement: Supplementary file 7 — Additional file 7: Table S6. Oligonucleotide and primer sequences. [file 13059_2022_2656_MOESM7_ESM.pdf]

Table S6: Oligonucleotide and primer sequences.

RNA oligonucleotides used in melting experiments.

Sequence	Shortened Name	Description	Tm (°C)
5’ GAGACGCGCUUUGUCUC	*ZR3* REF	17 nt portion of AT3G54826 reference sequence	67.3
5’ GAGACGCGCUUUAUCUC	*ZR3* ALT	17 nt portion of AT3G54826 alternative sequence	48.8
5’ GCGUUUGAGAGCAGAGCACTCGA	*CGR3* REF	23 nt portion of AT5G65810 reference sequence	32.7; 61.8
5’-GCGUUUGAGAACAGAGCACTCGA	*CGR3* ALT	23 nt portion of AT5G65810 alternative sequence	30; 51.7

Double stranded DNA gBlock sequences and their resulting *in vitro* transcribed RNA products. The SNP is bolded in red, the T7 promoter sequence is underlined, and nucleotides added to promote transcription of the sequences are bolded.

Sequence	Shortened Name	Description
5’ **GATCGT**TAATACGACTCACTATA**GG**CTCCCTCAGGTTG<br>TCAATGGCCGCTAGGTTACTTGCTTTGAGACGCGCTTT**G**TC<br>TCTTTTCAGCAACCAACAACATCGTTTTCCTTTGTCTCAAG<br>TCTCAACA	*ZR3* reference gBlock	gBlock sequence ordered from IDT for AT3G54826 reference
5’ **GATCGT**TAATACGACTCACTATA**GG**CTCCCTCAGGTTG<br>TCAATGGCCGCTAGGTTACTTGCTTTGAGACGCGCTTT**A**TC<br>TCTTTTCAGCAACCAACAACATCGTTTTCCTTTGTCTCAAG<br>TCTCAACA	*ZR3* alternative gBlock	gBlock sequence ordered from IDT for AT3G54826 alternative
5’ **GATCGT**TAATACGACTCACTATAGGCAAGAAGTCTTAT<br>TGTTAGTTAAATGCGCAGAACTAAAACGGCCGCGTTTGAG<br>A**G**CAGAGCACTCGATAATTAACACTAGGGACGAAGAAGC<br>TCTCGTTTAGTAAATTTTAGC	*CGR3* reference gBlock	gBlock sequence ordered from IDT for AT5G65810 reference
5’ **GATCGT**TAATACGACTCACTATAGGCAAGAAGTCTTAT<br>TGTTAGTTAAATGCGCAGAACTAAAACGGCCGCGTTTGAG<br>A**A**CAGAGCACTCGATAATTAACACTAGGGACGAAGAAGC<br>TCTCGTTTAGTAAATTTTAGC	*CGR3* alternative gBlock	gBlock sequence ordered from IDT for AT5G65810 alternative
5’ **GG**CTCCCTCAGGTTGTCAATGGCCGCTAGGTTACTTGCT<br>TTGAGACGCGCTTT**G**TCTCTTTTCAGCAACCAACAACATCG<br>TTTTCCTTTGTCTCAAGTCTCAACA	*ZR3* reference RNA	AT3G54826 reference RNA sequence used in *in vitro* DMS experiments
5’ **GG**CTCCCTCAGGTTGTCAATGGCCGCTAGGTTACTTGCT<br>TTGAGACGCGCTTT**A**TCTCTTTTCAGCAACCAACAACATCG<br>TTTTCCTTTGTCTCAAGTCTCAACA	*ZR3* alternative RNA	AT3G54826 alternative RNA sequence used in *in vitro* DMS experiments

5' GGCAAGAAGTCTTATTGTTAGTTAAATGCGCAGAACTA AAACGGCCGCGTTTGAGA**G**CAGAGCACTCGATAATTAACA CTAGGGACGAAGAAGCTCTCGTTTAGTAAATTTTAGC	*CGR3* reference RNA	AT5G65810 reference RNA sequence used in *in vitro* DMS experiments
5' GGCAAGAAGTCTTATTGTTAGTTAAATGCGCAGAACTA AAACGGCCGCGTTTGAGA**A**CAGAGCACTCGATAATTAACA CTAGGGACGAAGAAGCTCTCGTTTAGTAAATTTTAGC	*CGR3* alternative RNA	AT5G65810 alternative RNA sequence used in *in vitro* DMS experiments

Primer sequences utilized to PCR amplify gBlocks and reverse transcribe RNA.

**Sequence**	**Description**
5' GATCGTTAATACGACTCACTATAG	Universal forward primer for gBlock PCR amplification
5' TGTTGAGACTTGAGACAAAGG	AT3G54826 (*ZR3*) reverse primer used for both gBlock PCR amplification and labeled with γATP for reverse transcription
5' GCTAAAATTTACTAAACGAGAGC	AT5G65810 (*CGR3*) primer for gBlock PCR amplification and labeled with γATP for reverse transcription
