# Supplementary material for: Experimental demonstration and pan-structurome prediction of climate-associated riboSNitches in Arabidopsis
Source: Genome Biol. 2022 Apr 19;23:101. doi: 10.1186/s13059-022-02656-4 (PMC9017077; doi:10.1186/s13059-022-02656-4)
Supplement: Supplementary file 8 — Additional file 8. Review history. [file 13059_2022_2656_MOESM8_ESM.docx]

Review History

**First round of review**

**Reviewer 1**

**Were you able to assess all statistics in the manuscript, including the appropriateness of statistical tests used?**

No, I do not feel adequately qualified to assess the statistics.

**Comments to author:**

Ferrero-Serrano and co-authors correlated RNA-structure-altering polymorphisms (riboSNitches) in Arabidopsis plants with temperature acclimation. They identified and validated riboSNitches in two transcripts associated with plant continentality and temperature adaptation. The sequence/structure changes altered the abundance of these transcripts. Building on these results, the authors developed a new web tool called T-CLIM.

The manuscript presents a comprehensive study, is informative and well written. The aims of the study are clear and the major conclusions are supported by experimental data. The new web resource covering almost 3,000 Arabidopsis genomes is going to be a valuable tool for plant biologists.

Comments and questions.

1. Parts of figures 1 and 2 look (or even are) identical. In case 1B and 2B are identical, 1C/2C and 1E/2E will in turn also be identical, right? Then, Fig. 2 would probalby only need panels A, D, F and G.

2. Since Fig. 1B and 2B look very similar (or identical), I assume that all the continental plants marked with a red dot have a G to A riboSNitch in both ZR3 and CGR3? Isn't such a strict co-occurrence extremely surprising? Does it suggest a strict functional correlation between these genes?

3. Line 191: Did the author check for duplex formation experimentally, e.g. by native PAGE?

4. The results on the short RNA hairpins in Fig. 3 do not seem to contribute much to understanding the riboSNitch mechanism. The RNA structures of the variants in the longer and more authentic context (Fig. 4 and 5) have nothing in common with the simple hairpins.

In Fig. 3E, the melting behavior of the REF and ALT structures is almost identical. If that were the case in the context of the full-length transcript, the riboSNitch would probably not be able to affect transcript levels (Fig. 2) if no differences in melting or other structural changes occur.

5. Lines 399 to 418: Positive selection for temperature-associated SNPs in the heat shock-related gene ZR3 or in CGR3, which might be involved in cell wall stabilization, is conceivable. However, I find it counterintuitive that both riboSNitch reduce transcript abundance (Fig. 1) in heat-adapted plants. Wouldn't one expect the opposite?

Minor issues:

1. Line 223 and elsewhere. The gene designations are complicated. I suggest not switching back and forth between AT5G65810 and CGR3 but stay with CGR3 from line 155.

2. Line 194: Figure XA

3. 195: question mark after 22.5

4. Line 222: Figure XF

5. Line 491: incomplete sentence?

6. Line 797: Description of panel D is missing in the legend to figure 1.

7. Line 826: Panel D twice in legend to figure 3?

**Reviewer 2**

**Were you able to assess all statistics in the manuscript, including the appropriateness of statistical tests used?**

Yes, and I have assessed the statistics in my report.

**Comments to author:**

My apologies to the authors for the delay in reviewing their manuscript.

In this novel paper by Ferrero-Serrano and colleagues the authors present the discovery of temperature dependent riboSNitches in plants associated with geographical location in plants. Although such RNA regulatory elements had been theorized in the past, this is the first experimental confirmation of their presence as far as this reviewer knows. Both temperature sensing RNA elements and riboSNitches have been previously characterized in eukaryotes and prokaryotes, but never a functional SNP that alters temperature sensing. This is therefore a significant finding and further demonstrates the important role of RNA structure in genetic regulation and genomics and as such warrants publication in Genome Biology. I have a couple minor concerns the authors should consider.

1.) The ZR3 riboSNitch is in a coding sequence, and as far as I can tell is synonymous? This is not explicitly said in the manuscript and probably should be in the figure legend too. The authors should also confirm this SNP is not creating a rare codon which could impact translation efficiency. Also, the SNP is near an exon intron junction and the authors should confirm this is not causing allele-specific alternative splicing, by for example analyzing allele-specific RNA-seq in a heterozygous plant.

2.) The GCR3 riboSNitch is in the 5' UTR so will not affect protein sequence, however the authors should ensure it does not create a uORF. They should also check it does not lead to alternative transcription start site usage either by 5' RACE in both homozygous alleles, or if there is sufficient coverage in an heterozygous allele-specific RNA-seq experiment.

3.) This could be simply due to the pdf quality of the reviewer manuscript, but the probability plots on Figures 2 and 3 are low resolution, and the authors should try and embed the vectorized form of the plot in their final proofs.

4.) It is not clear if the DMS data shown in Figure 2 and 3 is averaged over replicates or a single replicate. If it is averaged over replicates, the authors should consider using a "skyline" plot with error bars to more clearly show where statistical differences exits. Also, they authors should label their x-axes with genomic coordinates.

**Authors Response**

**Point-by-point responses to the reviewers’ comments:**

**Response to the Reviewers’ Comments**

*We thank the two reviewers for the insightful comments and have revised the manuscript in accordance with their comments, as detailed in the point-by-point reply to their comments below, including particular attention paid to the points highlighted by the editor in her decision letter.*

**Point-by-point reply to the reviewers’ comments**

Reviewer #1:

Ferrero-Serrano and co-authors correlated RNA-structure-altering polymorphisms (riboSNitches) in Arabidopsis plants with temperature acclimation. They identified and validated riboSNitches in two transcripts associated with plant continentality and temperature adaptation. The sequence/structure changes altered the abundance of these transcripts. Building on these results, the authors developed a new web tool called T-CLIM.

The manuscript presents a comprehensive study, is informative and well written. The aims of the study are clear and the major conclusions are supported by experimental data. The new web resource covering almost 3,000 Arabidopsis genomes is going to be a valuable tool for plant biologists.

*Reply: We appreciate the reviewer’s positive comments regarding our research and the value of our web tool to the scientific community.*

Comments and questions.

1. Parts of figures 1 and 2 look (or even are) identical. In case 1B and 2B are identical, 1C/2C and 1E/2E will in turn also be identical, right? Then, Fig. 2 would probalby only need panels A, D, F and G.

*Reply: We thank the reviewer for pointing this out. Indeed, there was an accidental duplication of Figure 2B as Figure 1B. The correct Figure 1B has now been supplied.*

*The reviewer is also correct that Figures 1E and 2E are identical, but this has been done deliberately for parallel structure of the two figures and for ease of comparison with the rest of each figure. In the revised manuscript, we explicitly point this out in the Fig. 2 legend.*

*Please note that we also changed the colors in Figures 1E and 2E in the revised version: this does not change the content but was done because red and blue colors have a different meaning in the rest of Figure 1/Figure2 than in panels 1E and 2E and so our use of red and blue in the original Figures 1E and 2E could be confusing to the reader.*

2. Since Fig. 1B and 2B look very similar (or identical), I assume that all the continental plants marked with a red dot have a G to A riboSNitch in both ZR3 and CGR3? Isn't such a strict co-occurrence extremely surprising? Does it suggest a strict functional correlation between these genes?

*Reply: We note that even after the correction, Figs. 1B and 2B are still similar. This is because the minor alleles are in fairly high frequency for both ZR3 and CGR3 (11.3% and 9.6%, respectively), so this numerically favors co-variation. This co-variation would have been a concern if the variants were associated by linkage disequilibrium. However, ZR3 and CGR3 are on different chromosomes. In addition, the SNVs of both genes associate with many of the same climate parameters, which favors a similar distribution of both alleles across the distribution range of the species. The two genes are probably not functionally related because one encoded protein localizes to the Golgi apparatus (CGR3) while the other (ZR3) localizes to mitochondria.*

*3. Line 191: Did the author check for duplex formation experimentally, e.g. by native PAGE?*

*Reply: The reviewer is correct that duplex formation can be an issue in melts. The Bevilacqua lab has evaluated duplex formation with native PAGE in other studies (Heinicke et al., 2009), e.g. Fig. 2 and Fig. 7 in Heinicke et al., 2009. However, native gels work best with extensive base-pairing to slow down exchange in the gel, while the hairpins studied herein are only short sequences with limited base-pairing. As such, native PAGE is not the ideal assay for this study. To address duplex formation experimentally, we performed our melt assays over a range of RNA concentrations. If duplex formation occurs, the melts at higher concentrations will show a different melting profile from those at low concentrations. As described below, this assay worked because we found duplex formation in one of our sets of RNAs.*

*There was no indication of duplex formation for ZR3 reference (REF) or alternative (ALT) sequences in our original melts, a conclusion that remained unchanged when we repeated the ZR3 melts. Both the new and the original data for ZR3 indicate a lack of RNA concentration dependence to the Tm (Additional File 2A,B), i.e. the data are not obfuscated by duplex formation. Accordingly, we have not changed our description of the ZR3 melt data in the main text, nor have we changed the supporting melt data found in Additional File 2 from that found in the original submission. For Fig. 3, although the conclusions remained unchanged, our new melt runs were slightly less noisy and so have been substituted in Fig. 3B,C) in the revised manuscript.*

*For CGR3, the results were different. As reported in the original submission, two melting transitions were observed for both the REF and ALT sequences (see original Fig. 3E, F). Melts in our initial submission did show weak evidence of duplex formation for the first melting transition, as indicated by a right-shift to a slightly higher temperature in the first derivative peak (temperature range of 5 ºC – 45 ºC) upon going from 1.5 to 4.5 mM RNA concentration. We therefore conducted new melts, including an even higher RNA concentration, going up to 14.2 mM CGR3 RNA, where we used a short pathlength cuvette (0.1 cm vs. 1 cm pathlength) to keep in Beer’s law. Melts of both the REF and ALT sequence at this high RNA concentration showed a further right-shift of the first transition, confirming that the low temperature transition reflects melting of a duplex (see Additional File 2C,D); note that extrapolation to biologically low concentrations of RNA indicates the duplex won’t form at all in vivo. Importantly, the higher temperature transition revealed that first derivative curves from all three RNA concentrations overlay (Additional File 2E,F), indicating absence of duplex for this transition. In other words, the data are consistent with the RNA forming a self-complementary duplex at high RNA concentrations that melts out into a hairpin by ~40 ^o^C, which then melts to an unfolded conformation with T_M_’s near ~60 ^o^C and ~50 ^o^C for the REF and ALT, respectively. The first transition would be absent at biologically low concentrations of RNA (because it is from the unpairing of a duplex), while the second transition would be present (because it is from the unfolding of a hairpin). In the revised manuscript, we have replaced the original CGR3 data in Figure 3E,F and Additional File 2C,D with our new data (which were less noisy) and have also added first derivative analyses in Additional File 2 (see panels E,F). We discuss these complexities in text revisions on lines 221-229.*

*Importantly, duplex formation is not an issue in the interpretation of the gel-based structure probing data of Figs. 4 and 5, as those experiments were performed at a 2.5-fold (ZR3) to 5-fold (CGR3) lower concentration than the lowest concentrations used in the melt experiments. (Use of these low concentrations is not feasible in melt experiments as the noise in the melt assays at such low concentrations would overwhelm the true signal.)*

*A major point is that ZR3 is a conditional riboswitch because the folds of the REF and ALT in the longer construct (~100 nt) are nearly identical at 20 ^o^C (Fig. 4D) but become different at 37 ^o^C (Fig. 4E), while CGR3 may be a persistent riboswitch because the fold of the REF and ALT in the longer construct (~100 nt) are already different at 20 ^o^C (Fig. 5D); these folds become even more different at 37 ^o^C (Fig. 5E), suggestive of conditionality as well. We refer the referees to text on lines 378-380.*

4. The results on the short RNA hairpins in Fig. 3 do not seem to contribute much to understanding the riboSNitch mechanism. The RNA structures of the variants in the longer and more authentic context (Fig. 4 and 5) have nothing in common with the simple hairpins. In Fig. 3E, the melting behavior of the REF and ALT structures is almost identical. If that were the case in the context of the full-length transcript, the riboSNitch would probably not be able to affect transcript levels (Fig. 2) if no differences in melting or other structural changes occur.

*Reply: We performed additional analyses, the results of which address the reviewer’s statement that “The RNA structures of the variants in the longer and more authentic context (Fig. 4 and 5) have nothing in common with the simple hairpins.” Specifically, we folded the longer sequences that were used for the gel-based assays of Figs. 4 and 5 in three different ways: a) in silico, meaning with no experimental restraints; b) in vitro, meaning with the experimental restraints derived from our in vitro gel-based structure-probing data of Figs. 4, 5 and; c) in vivo, meaning for the REF sequences, with experimental restraints derived from our published in vivo Structure-seq data (Tack et al., 2020). Structures were predicted using RNAstructure (Reuter and Matthews, 2010) following our pipeline as established in our Nature publication (Ding et al., 2014). As illustrated below (Reviewer Figure 1, below), the short RNA hairpins are predicted to be present in the in silico, in vitro, and in vivo structures for the longer REF sequences for both ZR3 and CGR3. Therefore, contrary to the reviewer’s presumption, there is commonality between the simple hairpins and the longer sequences.*

*Reviewer Figure 1. RNA secondary structure predictions of a ZR3 and CGR3 at 20 ^o^C. (A-C) 103 nucleotide portion of the ZR3 reference sequence and (D-F) 115 nucleotide portion of the CGR3 reference sequence were folded at 20 °C using RNAStructure. The arrow indicates the location of the SNP in the short RNA oligonucleotides used in the UV-detected melt experiments. The predicted melt structures of the short RNA oligonucleotides are provided for reference as insets in panels B and E. Blue highlights indicate the position of the short RNA oligonucleotides within the sequences analyzed by gel-based structure probing. (A, D) The leftmost structures were predicted and folded in silico at 20 °C, using RNAstructure. The remaining structures were predicted using RNAstructure upon incorporation of restraints from (B, E) in vitro DMS reactivity data and (C, F) in vivo DMS reactivity data. In vitro DMS reactivity was derived from the two sequencing gels provided in Additional File 3, and in vivo DMS reactivity was derived from a published dataset from Arabidopsis plants grown at 21 ^o^C (Tack et al., 2020). The free energy at 20 ^o^C (in kcal/mol) for each folded RNA sequence was reported by the RNAStructure software.*

*To address the reviewer’s suggestion that the riboSNitch is not affecting transcript levels in Fig. 2 because the melting behavior of the REF and ALT for the small RNA hairpin is “almost identical,” we emphasize that the melting behavior is not almost identical. For ZR3, the T_M_s for the REF and ALT differ by ~30 ^o^C (Fig. 3C compare peaks), and for CGR3, they differ by ~15 ^o^C (Fig. 3F; compare peak 2, which is for the hairpin (see above)).*

5. Lines 399 to 418: Positive selection for temperature-associated SNPs in the heat shock-related gene ZR3 or in CGR3, which might be involved in cell wall stabilization, is conceivable. However, I find it counterintuitive that both riboSNitch reduce transcript abundance (Fig. 1) in heat-adapted plants. Wouldn't one expect the opposite?

*Reply: Transcript abundances in the published RNA-seq datasets were only at one temperature, 22 ^o^C, so the reviewer may be extrapolating from the climate conditions in Figs. 1 and 2. However, it is important to note that these SNPs actually are not correlated with heat – they are correlated with temperature seasonality, i.e. the temperature range over the year, which is greater higher inland than at the coast. When we assess these SNPs’ correlations with the maximum temperature of the warmest month, we do not find significant associations (Reviewer Figure 2 below; note that R^2^ values are essentially zero).*

*However, the reviewer’s comment did point out that we were remiss in not describing in the Discussion possible functions of these genes over a range of temperatures. As noted in the original text, ZR3 is a functional ortholog of a yeast protein that chaperones Hsps. However, one limitation of the cited study showing that ZR3 could substitute for its yeast ortholog in conferring tolerance to heat stress, is that only the reference version and not the alternative version of ZR3 was tested. Moreover, Hsps in plants, intriguingly, have been implicated in both heat and cold tolerance. This is consistent with ZR3’s association with temperature variation. We have accordingly noted this in our revised text: “Our results suggest that it would be of great interest in future studies to compare these variant ZR3 sequences in yeast complementation assays over a temperature cline, especially given that plant Hsps are also associated with cold tolerance (Ko et al., 2017; Heinicke et al., 2009), and that ZR3 variation is not associated with absolute temperature parameters, but rather with temperature variation (Fig. 1).”*

*For CGR3, very little work has been published on this pectin methylesterase. However, further investigation of the literature found evidence of a role for pectin methylesterification in acclimation to both heat and cold stress, consistent with the association of CGR3 variants with seasonality. Accordingly, we now write in the revised version of the text:*

*“The extent of methylesterification has been shown to affect tolerance of temperature extremes. Null mutants of the pectin methylesterase PME34 show increased susceptibility to heat stress but, interestingly, acclimation to cold also has been associated with increased pectin methylesterification (Baldwin et al., 2014). Our results suggest that it could be informative to assess variants of CGR3 for their impact on the production of CGR3 protein, CGR3 enzymatic activity, and relative tolerance of temperature extremes.”*

***Reviewer Figure 2. Allele distributions and transcript abundance in a synonymous SNP in ZR3 and in a SNP in the 5’ UTR of CGR3 are not correlated with the maximum temperature of the warmest month.*** *Regressions are calculated using the transcript abundance data from Kawakatsu et al. (2016) in the combined set of accessions (both major and minor alleles). Climate variables are from the WorldClim 2.1 database.*

*In addition, for both genes, across accessions transcripts containing the ALT SNP have more constant, albeit lower, expression over a range of seasonalities, whereas transcripts containing the REF SNP vary more in expression with seasonality (cf. Fig. 1F, G and Fig. 2F, G). Thus, the ALT riboSNitch may be maintaining stability of expression over a broad temperature range; these ALT SNPs are the ones enriched in accessions that experience a greater temperature range (greater seasonality).*

Minor issues:

1. Line 223 and elsewhere. The gene designations are complicated. I suggest not switching back and forth between AT5G65810 and CGR3 but stay with CGR3 from line 155.

2. Line 194: Figure XA

3. 195: question mark after 22.5

4. Line 222: Figure XF

5. Line 491: incomplete sentence?

6. Line 797: Description of panel D is missing in the legend to figure 1.

7. Line 826: Panel D twice in legend to figure 3?

*Reply: We appreciate the reviewer’s noting of these minor issues and have corrected all of them in the revised manuscript.*

Reviewer #2:

My apologies to the authors for the delay in reviewing their manuscript.

In this novel paper by Ferrero-Serrano and colleagues the authors present the discovery of temperature dependent riboSNitches in plants associated with geographical location in plants. Although such RNA regulatory elements had been theorized in the past, this is the first experimental confirmation of their presence as far as this reviewer knows. Both temperature sensing RNA elements and riboSNitches have been previously characterized in eukaryotes and prokaryotes, but never a functional SNP that alters temperature sensing. This is therefore a significant finding and further demonstrates the important role of RNA structure in genetic regulation and genomics and as such warrants publication in Genome Biology.

*Reply: We appreciate the reviewer’s observations that our findings are “novel” and “significant” and “warrant publication in Genome Biology.” Below, we address his/her minor concerns.*

I have a couple minor concerns the authors should consider.

1.) The ZR3 riboSNitch is in a coding sequence, and as far as I can tell is synonymous? This is not explicitly said in the manuscript and probably should be in the figure legend too.

*Reply: It is indeed synonymous. This was noted in the Figure 1 legend and in the Discussion. To make this more evident, in the revised manuscript, we now point this out much earlier, in the first paragraph of the Results section: “In ZR3 the variant is a synonymous substitution in the coding sequence while in CGR3 the variant is located in the 5’ UTR.”*

The authors should also confirm this SNP is not creating a rare codon which could impact translation efficiency.

*Reply: This riboSNitch is in a codon coding for leucine. As provided at https://www.kazusa.or.jp/codon/cgi-bin/showcodon.cgi?species=3702, with reference to Nakamura et al. (2000), in Arabidopsis, the frequency (per 1000 CDS nucleotides) of the REF codon UUG is 20.9/1000. The ALT UUA codon frequency is somewhat less, at 12.7/1000. There are four additional codons for leucine, with frequencies of 24.1, 16.1, 9.9, and 9.8. As a point of reference, the rarest non-stop codon is CGC (arginine), with a frequency of 3.8. Given these comparisons, the ALT codon is not rare, although it is less frequent than the REF codon. However, we calculated that among the 64 codons of ZR3, the REF and ALT codons are equally present, both at a frequency of 12.54/1000, suggesting that there is no codon usage bias between these two codons within ZR3.*

*We further addressed this question by calculating the codon bias index (Bennetzen and Hall, 1982) for all codons in ZR3. We find (Reviewer Figure 3) that the REF and ALT Leu codons do not differ in their codon bias index (CBI), indicating that there is no differential bias in codon usage between the REF and ALT codons arising from this SNV.*

***Reviewer Figure 3****. The distribution of the Codon Bias Index (Bennetzen and Hall, 1982) for all triplets in ZR3 reveals that the two different triplets coding for leucine resulting from the synonymous SNV do not differ in their CBI, with both displaying a low CBI (REF highlighted with a blue line, and ALT with a red line). CBI measures the extent that preferred codons are used in a gene. The score is between 1 (only preferred codons are used) and -1 (non-preferred codons are favored). CBI values near zero are considered to be unbiased.*

*With regard to translation efficiency, a systematic analysis by the Plotkin lab (Kudla et al., 2009) study found that mRNA structural effects on translation efficiency are strongly associated with a window from -4 to +37 nucleotides of the translation start site. The ZR3 SNV, at position +125, is not within this window. However, given that RNA structural effects of a riboSNitch can propagate away from its site, structural changes associated with the ZR3 riboSNitch may still affect ZR3 protein production.*

*We also performed further analyses to address the possibility that this SNV is affecting translation. Our colleague, Prof. Edward O’Brien, has developed a method to calculate codon translation rates from Ribo-Seq data (Sharma et al., 2019). Unfortunately, ZR3 was not present in multiple Arabidopsis Ribo-Seq datasets that we queried (Bazin et al., 2017; Branco-Price et al., 2008; Juntawong et al., 2014; Kawaguchi & Bailey-Serres, 2005). On the advice of Dr. O’Brien, we instead calculated the translation rates for each of the codons present in the ZR3 REF and ALT sequences using Ribo-Seq data from yeast (Nissley et al., 2016), applying individual codon translation rates as calculated using the method of Sharma et al. (2019). As can be seen in Reviewer Figure 4 below, the two alternative triplets coding for Leu in our REF and ALT sequences have very similar predicted translation times. This additional analysis also does not support the hypothesis that this SNV is affecting translational efficiency; however, because this analysis is based on yeast data, we have chosen not to include it in the revised manuscript.*

***Reviewer Figure 4.*** *The riboSNitch causing a synonymous change in the Leu codon in ZR3 is not expected to produce a significant change in the translation rates of Leu. This figure shows the distribution of the mean translation time for all triplets in ZR3 upon applying the methods of Sharma et al. (2019) to Ribo-Seq data from yeast (Nissley et al., 2016).*

*Accordingly, with regard to the two topics of codon usage and translational efficiency, we now write in the text:*

*Translation can also be impacted by synonymous SNPs either by virtue of differences in codon usage or by structural effects (Sharma & O’Brien 2018; Yang et al., 2014). Both the REF and ALT codons (for leucine) at this position are at a frequency of 12.54/1000 in the ZR3 transcripts in this population, suggesting that natural selection at this site does not reflect differential codon usage bias for REF vs. ALT. mRNA structural effects on translation efficiency have been strongly associated with a window from -4 to +37 of the translation start (Kudla et al., 2009). The ZR3 SNV, at position +125, is not within this window. However, given that RNA structural effects of a riboSNitch can propagate away from its site, structural changes associated with the ZR3 riboSNitch may still affect ZR3 protein production.*

Also, the SNP is near an exon intron junction and the authors should confirm this is not causing allele-specific alternative splicing, by for example analyzing allele-specific RNA-seq in a heterozygous plant.

*Reply: Whether this SNP may be regulating alternative splicing is an important question. This SNP is not located at an intron-exon junction (it is 43 nucleotides away from the 5’ end of the downstream intron). The reviewer proposes an interesting experiment with heterozygotes, but a limitation of this experiment is that it would be assessing the function of the SNP in a genetic background derived from only two accessions. Instead, we have performed a bioinformatic analysis of the 558 Eurasian accessions that have published RNA-seq datasets (Kawakatsu et al., 2016). Our analyses of these RNA-seq data show no differences in alternative splicing between the accessions harboring the major allele and the accessions harboring the minor allele. Therefore, the hypothesis that the SNP confers alternative splicing is not supported. In other words, based on these wet bench data from 558 accessions, we conclude that this SNP is not regulating alternative splicing (Additional File 7). We now note this in the text:*

*“We additionally evaluated the transcriptomes of the 558 Eurasian accessions (Kawakatsu et al., 2016) for a potential effect of this SNV in alternative splicing but found no differences in splice site choice between accessions harboring the reference vs. the alternative SNP.”*

2.) The GCR3 riboSNitch is in the 5' UTR so will not affect protein sequence, however the authors should ensure it does not create a uORF.

*Reply: We have assessed the REF and ALT 5’ UTR sequences of CGR3 for the canonical AUG start codon and for stop codons, and none of these codons are present. In addition, CGR3 is not found in a 2014 database of Arabidopsis genes that contain uORFs (von Arnim et al., 2014) (see Supplemental Database 1 of that paper). However, absence of canonical start and stop codons is not completely definitive, as uORFs can utilize non-canonical start codons in the 5’ UTR and stop codons in the main ORF that are in-frame with the uORF. Therefore, we also assessed the possible presence of a uORF in CGR3 based on a more recent bioinformatic analysis that includes the possibility of non-canonical start codons and alternative stop codons. In this analysis from van der Horst et al. (2019), CGR3 does not appear in the list of uORF-containing genes identified by their pipeline, even when the most stringent filtering steps of their pipeline are omitted (see Supplemental Data 1 of that paper). Moreover, JBrowse does not annotate any uORFs for CGR3, citing a personal communication from Prof. Julia Bailey-Serres, who has performed extensive ribosome profiling studies. On the other hand, a recent analysis (Li & Liu, 2020) of one proteomics dataset (Willems et al., 2017) suggests that CGR3 has an alternative translation start site consisting of an AUC that is 467 nt upstream of the canonical AUG translation start. Our CGR3 SNV is 125 nt upstream of this CUU.*

*In summary, there is no evidence that the CGR3 ALT SNP itself introduces a translation start site. Because there is no consensus in the current literature as to whether CGR3 contains a uORF, we conclude that it would be unduly speculative to suggest that this riboSNitch influences the position of translation initiation.*

They should also check it does not lead to alternative transcription start site usage either by 5' RACE in both homozygous alleles, or if there is sufficient coverage in an heterozygous allele-specific RNA-seq experiment.

*Reply: We again turned to the dataset of 558 Eurasian transcriptomes for a comprehensive evaluation of this interesting hypothesis. For the 558 Eurasian accessions that had published RNA-seq datasets, we found no evidence of a different transcription start site in the accessions that harbor the major allele of CGR3 vs. the accessions that harbor the minor allele of CGR3. This analysis is provided in (Additional File 7).*

With regard to the possibility of an alternative transcription start site, we now note in the revised text: “*While variants in 5’ UTRs could theoretically affect transcription start sites (TSS) our analyses (Additional File 7B) of the transcriptomes analyzed herein produced no evidence of different TSS for CGR3 reference vs. alternative sequences.”*

3.) This could be simply due to the pdf quality of the reviewer manuscript, but the probability plots on Figures 2 and 3 are low resolution, and the authors should try and embed the vectorized form of the plot in their final proofs.

*Reply: Thank you for pointing this out. This comment probably refers to Figures 4B and 5B which are probability plots. We have improved the resolution of these plots in the newly uploaded figures.*

4.) It is not clear if the DMS data shown in Figure 2 and 3 is averaged over replicates or a single replicate. If it is averaged over replicates, the authors should consider using a "skyline" plot with error bars to more clearly show where statistical differences exits.

*Reply: The DMS data are presented in Figures 4 and 5. These data are representative rather than averaged figures, so we have not changed these plots. We have run several more gels and consistently see differences between the REF and ALT sequences.*

Also, they authors should label their x-axes with genomic coordinates.

*Reply: This is an excellent suggestion. However, because one of these genes is on the reverse strand, we decided that it would be more informative to include this information in the figure legend, where we have space to indicate this.*

***References***

*Baldwin, L, Domon, JM, Klimek, JF, Fournet, F, Sellier, H, Gillet, F, ... & Rayon, C:.* ***Structural alteration of cell wall pectins accompanies pea development in response to cold.*** *Phytochemistry 2014,* ***104:*** *37-47.*

*Bazin, J, Baerenfaller, K, Gosai, SJ, Gregory, BD, Crespi, M, & Bailey-Serres, J:* ***Global analysis of ribosome-associated noncoding RNAs unveils new modes of translational regulation.*** *Proc Natl Acad Sci USA 2017,* ***114:*** *E10018-E10027.*

*Branco‐Price, C, Kaiser, KA., Jang, CJ., Larive, CK., & Bailey‐Serres, J:* ***Selective mRNA translation coordinates energetic and metabolic adjustments to cellular oxygen deprivation and reoxygenation in Arabidopsis thaliana.*** *Plant J 2008,* ***56:*** *743-755.*

*Bennetzen, JL., & Hall, BD:* ***Codon selection in yeast.*** *J Biol Chem 1982,* ***257:*** *3026-3031.*

*Ding, Y, Tang, Y, Kwok, CK, Zhang, Y, Bevilacqua, PC, & Assmann, SM:* ***In vivo genome-wide profiling of RNA secondary structure reveals novel regulatory features.*** *Nature 2014,* ***505:*** *696-700.*

*Heinicke, LA, Wong, CJ., Lary, J, Nallagatla, SR., Diegelman-Parente, A, Zheng, X, ... & Bevilacqua, PC:* ***RNA dimerization promotes PKR dimerization and activation.*** *J Mol Biol 2009,* ***390:*** *319-338.*

*Juntawong, P., Girke, T., Bazin, J., & Bailey-Serres, J:* ***Translational dynamics revealed by genome-wide profiling of ribosome footprints in Arabidopsis****. Proc Natl Acad Sci USA 2014,* ***111:*** *E203-E212.*

*Kawaguchi, R, & Bailey-Serres, J:* ***mRNA sequence features that contribute to translational regulation in Arabidopsis.*** *Nucleic Acids Res 2005,* ***33:*** *955-965.*

*Kawakatsu T, Huang SSC, Jupe F, Sasaki E, Schmitz RJ, Urich MA, Castanon R, Nery JR, Barragan C, He YP, et al:* ***Epigenomic Diversity in a Global Collection of Arabidopsis thaliana Accessions.*** *Cell 2016,* ***166:****492-505.*

*Ko, E, Kim, M, Park, Y, & Ahn, YJ:* ***Heterologous expression of the carrot Hsp17. 7 gene increased growth, cell viability, and protein solubility in transformed yeast (Saccharomyces cerevisiae) under heat, cold, acid, and osmotic stress conditions.*** *Curr Microbiol 2017,* ***74:*** *952-960.*

*Kudla, G, Murray, AW, Tollervey, D, & Plotkin, JB:* ***Coding-sequence determinants of gene expression in Escherichia coli.*** *Science 2009,* ***324:*** *255-258.*

*Li, YR., & Liu, MJ:* ***Prevalence of alternative AUG and non-AUG translation initiators and their regulatory effects across plants.*** *Genome Res 2020,* ***30:****1418-1433.*

*Nakamura, Y, Gojobori, T, & Ikemura, T:* ***Codon usage tabulated from international DNA sequence databases: status for the year 2000.*** *Nucleic Acids Res 2000,* ***28:*** *292-292.*

*Nissley, DA, Sharma, AK, Ahmed, N, Friedrich, UA, Kramer, G, Bukau, B, & O’Brien, EP:* ***Accurate prediction of cellular co-translational folding indicates proteins can switch from post-to co-translational folding.*** *Nat Commun 2016,* ***7:*** *1-13.*

*Reuter, JS, & Mathews, DH:* ***RNAstructure: software for RNA secondary structure prediction and analysis.*** *BMC Bioinformatics 2010,* ***11:*** *1-9.*

*Sharma, AK, & O’Brien, EP:* ***Non-equilibrium coupling of protein structure and function to translation–elongation kinetics.*** *Curr Opin Struct Biol 2018,* ***49:*** *94-103.*

*Sharma, AK, Sormanni, P, Ahmed, N, Ciryam, P, Friedrich, UA, Kramer, G, & O’Brien, EP:* ***A chemical kinetic basis for measuring translation initiation and elongation rates from ribosome profiling data****. PLoS Comput Biol 2019,* ***15:*** *e1007070.*

*Tack, DC, Su, Z, Yu, Y, Bevilacqua, PC, & Assmann, SM:* ***Tissue-specific changes in the RNA structurome mediate salinity response in Arabidopsis.*** *RNA 2020,* ***26:*** *492-511.*

*van der Horst, S, Snel, B, Hanson, J, & Smeekens, S:* ***Novel pipeline identifies new upstream ORFs and non-AUG initiating main ORFs with conserved amino acid sequences in the 5′ leader of mRNAs in Arabidopsis thaliana.*** *RNA 2019,* ***25:*** *292-304.*

*Von Arnim, AG., Jia, Q, & Vaughn, J N:* ***Regulation of plant translation by upstream open reading frames****. Plant Sc 2014,* ***214:*** *1-12.*

*Willems, P, Ndah, E, Jonckheere, V, Stael, S, Sticker, A, Martens, L, ... & Van Damme, P:* ***N-terminal proteomics assisted profiling of the unexplored translation initiation landscape in Arabidopsis thaliana.*** *Mol Cell Proteomics 2017,* ***16:*** *1064-1080.*

*Yang, JR, Chen, X, & Zhang, J:* ***Codon-by-codon modulation of translational speed and accuracy via mRNA folding.*** *PLoS Biol 2014,* ***12:*** *e1001910.*

**Second round of review**

**Reviewer 2**

The authors have done a commendable job of addressing my concerns, the paper can be accepted for publication.

It appears the authors have addressed all reviewer concerns adequately in my opinion and the paper in my opinion can be published.
